# Prognostic Value of Preoperative Inflammatory, Hematological, and Metabolic Blood Indices in Endometrioid-Type Endometrial Cancer: A Retrospective Cohort Study

**DOI:** 10.3390/medicina62091767

**Published:** 2026-09-14

**Authors:** Gorkem Ulger, Kasim Akay, Hamza Yildiz, Pelin Aytan, Sevki Goksun Gokulu, Tolgay Tuyan Ilhan

**Affiliations:** 1 Division of Gynecologic Oncology, Department of Obstetrics and Gynecology, Niğde Training and Research Hospital, 51100 Niğde, Turkey; 2 Department of Obstetrics and Gynecology, Düziçi State Hospital, 80600 Osmaniye, Turkey; kasimakay57@gmail.com; 3 Department of Obstetrics and Gynecology, Tarsus State Hospital, 33400 Mersin, Turkey; hamzayldz911@gmail.com; 4 Division of Hematology, Department of Internal Medicine, Faculty of Medicine, Mersin University, 33110 Mersin, Turkey; drpelinaytan@gmail.com; 5 Department of Obstetrics and Gynecology, Faculty of Medicine, Mersin University, 33110 Mersin, Turkey; sevkigoksungokulu@gmail.com (S.G.G.); tolgaytuyan@yahoo.com (T.T.I.)

**Keywords:** endometrial cancer, endometrioid carcinoma, inflammatory markers, prognosis, nucleated red blood cell

## Abstract

*Background and Objectives*: Peripheral blood indices have been proposed as prognostic biomarkers in endometrial cancer, but most reports include mixed histological subtypes, do not control for benign uterine pathology that independently alters hematological profiles, and evaluate these indices in isolation. We evaluated all three categories head-to-head in a histologically homogeneous endometrioid cohort and formally tested whether they add prognostic information beyond established clinicopathological variables. *Materials and Methods*: Of 195 patients operated for endometrioid-type endometrial cancer between 2018 and 2024, 142 were included; adenomyosis or leiomyoma on final histopathology, active infection and hematological disease were exclusion criteria. Fourteen preoperative blood-derived indices spanning the three categories were analyzed as continuous variables. A pre-specified clinical model (age category, grade, FIGO stage) was compared with models adding the biomarkers using likelihood-ratio tests, Harrell’s concordance index, time-dependent areas under the curve, bootstrap internal validation, and influence diagnostics. *Results*: Median follow-up was 52.5 months; 34 patients (23.9%) died and 5-year overall survival was 74.1%. None of the conventional inflammatory ratios were associated with survival (SII *p* = 0.893; all others *p* ≥ 0.26). In the multivariable model, histologic grade and NRBC% remained associated with survival (NRBC% HR 3.30 per 1%, 95% CI 1.09–9.97, *p* = 0.034). Adding NRBC% to the clinical model improved fit (likelihood-ratio *p* = 0.039) but not discrimination (ΔC 0.006, 95% CI −0.041 to 0.053, *p* = 0.814), and no index retained significance after Benjamini–Hochberg adjustment. The NRBC% association disappeared on dichotomization at the detection limit and could not be estimated once the five patients with values above 0.30% were excluded. *Conclusions*: In a predominantly early-stage endometrioid endometrial cancer cohort, preoperative blood-derived indices added no measurable discriminatory information beyond established pathological variables. The NRBC% association rests on very few observations at the upper end of its distribution and is hypothesis-generating only.

## 1. Introduction

Endometrial cancer is the most common gynecological malignancy in high-income countries, and its global incidence continues to rise, driven principally by increasing rates of obesity and an aging population [1]. In 2020, more than 417,000 new cases and nearly 100,000 deaths were attributed to endometrial cancer worldwide, ranking it second in incidence among female genital malignancies and third in mortality among gynecological cancers [2]. The endometrioid histotype accounts for the majority of cases and generally carries a more favorable prognosis than non-endometrioid subtypes; nevertheless, even within this comparatively favorable histological group, outcomes remain heterogeneous, and a subset of patients experience recurrence or death despite optimal surgical management [1,2].

The revised 2023 International Federation of Gynecology and Obstetrics (FIGO) staging system has refined prognostic discrimination by incorporating histological subtype, lymphovascular space invasion (LVSI), and molecular classification alongside traditional anatomical criteria [3,4]. However, these parameters are derived from surgical and pathological specimens and cannot be fully assessed before or during surgery, leaving a clinical need for inexpensive, readily available biomarkers capable of informing preoperative risk stratification and postoperative surveillance planning.

Systemic inflammation is increasingly recognized as a fundamental driver of tumor initiation, progression, and metastasis; activated neutrophils and neutrophil extracellular traps promote angiogenesis, suppress anti-tumor immunity, and facilitate distant spread [5]. This mechanistic link has prompted extensive study of inexpensive, routinely available peripheral blood-derived indices as prognostic biomarkers across solid tumors. Ratios such as the neutrophil-to-lymphocyte ratio (NLR), platelet-to-lymphocyte ratio (PLR), and systemic immune–inflammation index (SII) have shown associations with survival across gynecological malignancies, including ovarian, cervical, and endometrial cancer [6,7,8]. In endometrial cancer specifically, elevated pretreatment SII has been independently associated with inferior overall and progression-free survival in several cohorts, in some series outperforming NLR and PLR [8,9,10].

Beyond these established ratios, several less conventional hematological parameters have emerged as candidate prognostic markers. Nucleated red blood cells (NRBCs), virtually absent from the peripheral circulation of healthy adults, appear under conditions of marrow stress, hypoxia, and severe systemic inflammation, and their presence has been consistently linked to increased short-term mortality in critically ill and septic patients [11,12]. The inflammatory burden index (IBI), which combines C-reactive protein with the neutrophil-to-lymphocyte ratio, has demonstrated independent prognostic value in esophageal cancer [13,14], and circulating immature granulocytes (IGs), which reflect accelerated granulopoiesis under systemic inflammatory stress, have been associated with nodal metastasis in solid tumors such as breast cancer [15]. The fibrosis index based on four factors (FIB-4), originally developed to non-invasively grade hepatic fibrosis, has likewise shown prognostic associations in non-hepatic malignancies such as metastatic colorectal cancer [16], extending earlier oncological validation in hepatocellular carcinoma [17] and suggesting that it may capture systemic inflammatory or metabolic derangement beyond liver fibrosis per se.

A further limitation of the existing literature is the frequent, unaddressed coexistence of adenomyosis and uterine leiomyoma, both common benign entities associated with chronic local inflammatory activity. Adenomyosis is accompanied by measurable alterations in peripheral blood neutrophil parameters and inflammatory indices [18], and benign myometrial pathology produces hematological changes that overlap with those attributed to malignancy [19], and hemoglobin concentration and platelet count differ significantly across benign etiologies of abnormal uterine bleeding [20], so that the two contributions are difficult to disentangle from tumor-related changes. Comorbid conditions such as coronary artery disease (CAD), which shares overlapping chronic inflammatory pathways with malignancy, may likewise independently influence both circulating inflammatory indices and survival, yet are seldom incorporated into prognostic models of endometrial cancer [21].

Against this background, the present study was designed to evaluate the prognostic value of a comprehensive panel of preoperative inflammatory, hematological, and metabolic indices in a deliberately homogeneous cohort of patients with endometrioid-type endometrial cancer. To limit the confounding that has affected earlier work, the analysis was restricted to a single histological subtype and patients with histologically confirmed adenomyosis or uterine leiomyoma, active infection, or hematological disease were excluded. Twelve derived indices (NLR, PLR, MLR, LMR, SII, SIRI, PIV, GLR, APRI, FIB-4, IBI, and ICPI) were examined alongside NRBC% and IG% in the same patients under identical modeling assumptions, and every marker was treated as a continuous variable in order to avoid the optimism introduced by cohort-specific cut-off values. The primary objective was to determine which of these parameters, if any, retains an independent association with overall survival after adjustment for established clinicopathological prognostic factors, and thereby to clarify the conditions under which peripheral blood inflammatory, hematological, and metabolic indices provide usable prognostic information in endometrial cancer. A secondary objective was to test formally, by nested model comparison, whether any of these indices adds prognostic information beyond established clinicopathological variables.

## 2. Materials and Methods

### 2.1. Study Design and Setting

This retrospective study was conducted at the Gynecological Oncology Clinic, Department of Obstetrics and Gynecology, Mersin University Faculty of Medicine Hospital. The study protocol was approved by the Mersin University Clinical Research Ethics Committee (Decision No: 2024/532, 5 June 2024) and conducted in accordance with the Declaration of Helsinki.

**Figure 1:**
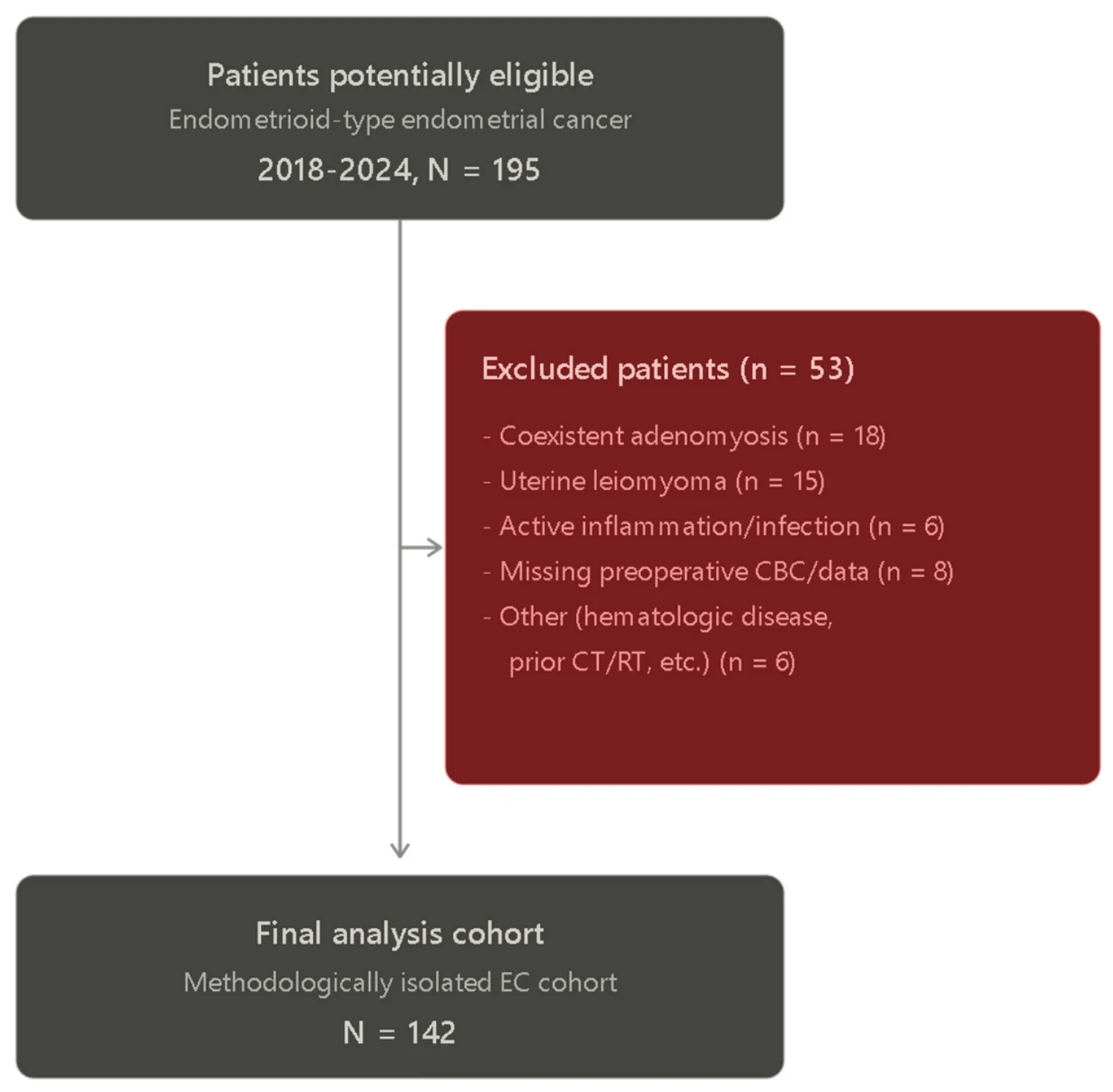
Flowchart of patient selection. Of 195 patients potentially eligible, 53 were excluded (coexistent adenomyosis *n* = 18; uterine leiomyoma *n* = 15; active inflammation or infection *n* = 6; missing preoperative complete blood count or incomplete records *n* = 8; other, including hematological disease and prior chemotherapy or radiotherapy, *n* = 6), leaving a final cohort of 142 patients with endometrioid-type endometrial cancer in whom benign uterine pathology had been excluded on final histopathology.

From a total of 195 patients who underwent surgery for endometrioid-type endometrial cancer between 2018 and 2024, 142 patients met the inclusion criteria and were included in the final analysis (Figure 1). The patient selection process involved careful screening to exclude confounding factors that could influence inflammatory marker interpretation.

### 2.2. Participants and Eligibility Criteria

The inclusion criteria comprised patients aged 18 years or older with histologically confirmed endometrioid-type endometrial cancer who underwent surgical staging with available preoperative blood count data within 7 days before surgery and complete follow-up data. The exclusion criteria were designed to eliminate potential confounders of inflammatory markers and included non-endometrioid histological subtypes, previous surgery for endometrial cancer, prior chemotherapy or radiotherapy, coexistent adenomyosis confirmed on final histopathology, coexistent uterine myomas (leiomyomas) confirmed on final histopathology, active inflammatory conditions or infections, hematological disorders, insufficient medical records, and age less than 18 years. Active inflammatory conditions or infections were defined as a documented clinical diagnosis of infection or an inflammatory disorder, antimicrobial or systemic corticosteroid or immunosuppressive therapy within the four weeks preceding surgery, or fever recorded at admission; no laboratory threshold was applied, since the blood indices under study were themselves the outcome of interest. Hematological disorders were defined as any documented diagnosis of a hematological malignancy, bone marrow disorder, hemolytic anaemia or coagulopathy, or ongoing treatment for such a condition.

The patient selection process began with 195 endometrioid-type endometrial cancer patients who underwent surgery during the study period. Following systematic application of exclusion criteria, patients with adenomyosis were excluded based on final histopathological examination. Patients with myoma uteri were excluded when final histopathology confirmed the presence of leiomyomas. Additional exclusions included patients with incomplete data, those with other exclusion criteria, resulting in the final study cohort of 142 patients.

### 2.3. Data Collection

Patient demographics, clinical characteristics, surgical details, and histopathological findings were retrospectively collected from medical records and the electronic patient registration system. All histopathological specimens were reviewed by experienced gynecologic pathologists at our institution using standardized criteria. Tumor grading followed World Health Organization classification guidelines. Age was categorized using a clinical threshold of 65 years, representing a widely accepted cut-off in gynecological oncology for defining geriatric patient populations. Adjuvant treatment and cause of death were also sought. Adjuvant therapy was recommended in accordance with the guidelines prevailing at the time of surgery, but a proportion of patients received their treatment at other institutions or did not attend, so the therapy actually delivered could not be verified for the whole cohort. Dates of death were obtained from the national civil registry, which records the date but not the cause of death, and the cause could not be established for patients who died outside our institution. Neither variable was therefore included in the analyses.

### 2.4. Blood Sampling and Derived Indices

Preoperative blood samples were obtained within 7 days before surgery after overnight fasting. Complete blood counts were analyzed in the hospital central laboratory on a Sysmex XN-1000 automated hematology analyzer (Sysmex Corporation, Kobe, Japan). NRBC% and IG% were reported automatically by this platform to one decimal place, the lowest non-zero value recorded in the cohort being 0.1% for both parameters. The scale printed against NRBC% on our laboratory report spans 0 to 5%; this is the reporting range of the percentage parameter and not a locally validated health-related reference interval, and the laboratory derives its reference limits from the absolute nucleated erythrocyte count rather than from the percentage. Because nucleated erythrocytes are normally absent from the peripheral blood of healthy adults [11,12], any detectable NRBC% is treated here as a low-level abnormal finding rather than as variation within a normal range. The reference interval for IG% was 0 to 0.6%. Complete blood count parameters including white blood cell (WBC) count, neutrophil count, lymphocyte count, monocyte count, platelet count (PLT), and hemoglobin (HB) were recorded; all cell counts are expressed as 10^3^/µL and platelet counts as 10^9^/L in the derived indices. Additional laboratory parameters including fasting plasma glucose (mg/dL), liver-function tests and, when available, C-reactive protein (CRP) and tumor markers were documented.

The blood-derived indices evaluated in this study fall into three biologically distinct categories, and were calculated using established formulas as follows.

Inflammatory and immune-cell indices:

- Neutrophil-to-Lymphocyte Ratio (NLR): Neutrophil count/Lymphocyte count.
- Platelet-to-Lymphocyte Ratio (PLR): Platelet count/Lymphocyte count.
- Monocyte-to-Lymphocyte Ratio (MLR): Monocyte count/Lymphocyte count.
- Lymphocyte-to-Monocyte Ratio (LMR): Lymphocyte count/Monocyte count.
- Systemic Immune–Inflammation Index (SII): Neutrophil count × Platelet count/Lymphocyte count.
- Systemic Inflammation Response Index (SIRI): Neutrophil count × Monocyte count/Lymphocyte count [22].
- Pan-Immune–Inflammation Value (PIV): Neutrophil count × Platelet count × Monocyte count/Lymphocyte count [23].
- Inflammatory Burden Index (IBI): C-reactive protein × Neutrophil count/Lymphocyte count.
- Inflammation-Combined Prognostic Index (ICPI): calculated as originally described [24], by scoring each of three components against its published threshold (LMR < 4.315, NLR ≥ 2.344 and PLR ≥ 212.01, each scored 1 and otherwise 0) and applying the published weights, ICPI = 2.9 × LMR score + 2.8 × NLR score + 2.8 × PLR score. The resulting index ranges from 0 to 8.5, and the threshold reported in the source publication is 2.9.

Metabolic and hepatic indices:

- Glucose-to-Lymphocyte Ratio (GLR): Glucose level (mg/dL)/Lymphocyte count [25].
- Aspartate Transaminase-to-Platelet Ratio Index (APRI): [(AST/upper limit of normal)/platelet count (10^9^/L)] × 100 [26]. The upper limit of normal for AST in our laboratory was 35 U/L.
- Fibrosis Index Based on Four Factors (FIB-4): (age [years] × AST)/(platelet count [10^9^/L] × √ALT).

Hematological parameters reported directly by the analyzer:

- Nucleated Red Blood Cell Percentage (NRBC%): nucleated erythrocytes expressed as a percentage of total nucleated cells, reported automatically by the analyzer.
- Immature Granulocyte Percentage (IG%): promyelocytes, myelocytes, and metamyelocytes expressed as a percentage of leukocytes, reported automatically by the analyzer.

### 2.5. Surgical Staging and Nodal Classification

All patients underwent comprehensive surgical staging according to established guidelines. The surgical approach included total hysterectomy and bilateral salpingo-oophorectomy. Lymph-node dissection decisions were based on intraoperative frozen section analysis and established criteria. Pelvic lymphadenectomy was performed for grade 1–2 tumors with diameters greater than 2 cm, myometrial invasion greater than 50%, or cervical stromal involvement. Para-aortic lymphadenectomy was performed for grade 3 tumors regardless of other factors, and for selected grade 1–2 cases based on intraoperative findings. The extent of lymph node dissection was determined by the surgical team based on individual patient characteristics and institutional protocols. Nodal status was classified strictly on histopathology and was not inferred from preoperative imaging or from intraoperative inspection. Patients were assigned to one of three categories, separately for the pelvic and the para-aortic nodal region: node-positive (pN1) when metastasis was histologically confirmed in a dissected node; node-negative (pN0) when the corresponding region was dissected and all retrieved nodes were free of metastasis; and not assessed (pNX) when the corresponding region was not dissected. No patient underwent para-aortic dissection without concurrent pelvic dissection, so the three surgical groups formed a nested hierarchy. Pelvic nodes were surgically assessed in 117 patients (82.4%), of whom 12 were pN1 and 105 pN0, while the 25 patients (17.6%) who underwent no lymphadenectomy were classified as pNX. Para-aortic nodes were surgically assessed in sixty-four patients (45.1%), of whom four were pN1 and sixty pN0, while the remaining seventy-eight patients (54.9%) were classified as pNX. Patients in whom a nodal region was not dissected were retained in a separate pNX category and were at no point described as node-negative or combined with pathologically confirmed pN0 cases. Preoperative pelvic and abdominal magnetic resonance imaging or computed tomography and intraoperative frozen section analysis of the primary uterine specimen (evaluating histologic grade, tumor size, and myometrial invasion) guided the surgical decision to omit pelvic and/or para-aortic lymphadenectomy in clinically low-risk cases; because that decision was itself determined by disease-extent assessment, pNX status reflects the surgical decision pathway rather than an estimate of true nodal status. Pelvic and para-aortic nodal status were therefore each analyzed as three-level categorical variables (pN0 as reference, pN1, pNX) rather than as dichotomous positive-versus-negative variables. All cases were staged according to the FIGO 2009 staging system, which was in use throughout the study period, and cases operated before 2023 were not restaged according to the FIGO 2023 revision. Molecular classification (POLE, mismatch repair, and p53 status) was not performed in this cohort, and lymphovascular space invasion was recorded as present or absent rather than as focal or substantial. Because the FIGO 2023 system assigns stage IIB on the basis of substantial LVSI and classifies grade 3 endometrioid carcinoma as an aggressive histological type, restaging without molecular data and without quantification of LVSI would have introduced systematic misclassification and was therefore not attempted.

### 2.6. Statistical Analysis

The study is reported in accordance with the STROBE statement for observational studies, and the completed checklist is provided as Supplementary Material. Descriptive statistics and the univariate and multivariable analyses presented in Table 1, Table 2, Table 3 and Table 4 were performed using IBM SPSS Statistics for Windows, version 22.0 (IBM Corp., Armonk, NY, USA); model comparison, discrimination, penalized regression, and assumption checking (Table 5) were performed in R version 4.6.1 (R Foundation for Statistical Computing, Vienna, Austria), using the survival, rms, timeROC, and coxphf packages. Continuous variables were expressed as mean ± standard deviation (SD) for normally distributed data or median with minimum and maximum values for non-normally distributed data. Categorical variables were presented as frequencies and percentages. The normality of continuous variables was assessed using the Kolmogorov–Smirnov and Shapiro–Wilk tests.

To avoid information loss and the statistical bias associated with arbitrary dichotomization, all preoperative laboratory parameters and hematological indices were analyzed as continuous variables in the primary survival models. Discriminative ability was instead assessed with methods appropriate for right-censored data, as described below.

Overall survival was defined as the interval from the date of surgery to death from any cause. Vital status was ascertained for every patient up to a common administrative censoring date of 1 June 2026; patients alive at that date were censored there, and no patient was censored earlier for loss to follow-up, since complete follow-up data were an inclusion criterion. Follow-up duration was therefore the interval from surgery to death or to 1 June 2026, and the median follow-up reported in Section 3.3 is the median of these observed follow-up times across the whole cohort rather than a reverse Kaplan–Meier estimate. Survival curves and cumulative survival rates were estimated using the Kaplan–Meier method, with between-group comparisons assessed using the log-rank test. Univariate Cox proportional hazards regression analysis was performed to identify demographic, clinicopathological, and continuous laboratory variables associated with OS. Variables demonstrating a significance level of *p* < 0.10 in the univariate analysis were considered candidates for multivariable modeling. To preserve model stability and avoid multicollinearity, overlapping hematological indices derived from shared blood-count components were restricted to a single representative marker per correlated pair (FIB-4 was retained over APRI, and MLR was retained over SIRI, based on the stronger univariate association). Surgical approach (laparotomy vs. laparoscopy) and extent of lymphadenectomy, although statistically associated with OS on univariate analysis, were excluded a priori from multivariable candidacy because these variables are direct consequences of preoperative and intraoperative disease-extent assessment (see Surgical Staging, above) rather than independent risk factors, introducing confounding by indication. Vaginal or parametrial metastasis were likewise excluded from multivariable candidacy despite univariate significance, as this subgroup comprised only two patients, precluding a stable effect estimate. The final candidate set (age category, coronary artery disease, histologic grade, distant metastasis, NRBC%, IG%, MLR, and FIB-4) was entered simultaneously into the multivariable Cox regression model (Enter method); no automated stepwise selection was performed. Two-tailed *p*-values < 0.05 were considered statistically significant.

The sample size was determined by the number of available eligible patients identified during the study period (N = 142). We evaluated model adequacy using the Events Per Variable (EPV) criterion. Vittinghoff and McCulloch have argued that the conventional rule of ten events per variable can be relaxed, and reported that EPV values in the range of five to nine were associated with only modest bias in estimated coefficients, confidence interval coverage and type I error [27]. With thirty-four events and nine parameters (accounting for the two-level grade contrast) in the primary multivariable model, the resulting EPV was 3.8, below this recommended range. A parallel sensitivity analysis restricted to the complete-case subset with available C-reactive protein data (*n* = 76, 16 events) was performed to evaluate the independent contribution of IBI, which could not be included in the primary model without a substantial reduction in sample size through listwise deletion (CRP/IBI data were available for 76 of 142 patients; 53.5%); this sensitivity model (age category, grade, and IBI) yielded an EPV of 4.0. Given that both EPV values fall below the conventionally recommended range, all multivariable findings in this study should be interpreted as exploratory and hypothesis-generating rather than confirmatory, and require external validation in larger cohorts.

Model stability and the incremental prognostic contribution of the biomarkers were assessed in a pre-specified analysis. A parsimonious clinical model was defined a priori on clinical grounds rather than on univariate *p*-values and comprised age category (≥65 versus <65 years), histologic grade (three levels), and FIGO stage (≥II versus I); with 34 events and four parameters, its events-per-variable ratio was 8.5. NRBC% was then added as a continuous term (five parameters, EPV 6.8), and a further exploratory model added IG%, MLR, and FIB-4 (eight parameters, EPV 4.3). The incremental contribution of the biomarkers was assessed by likelihood-ratio tests comparing nested models. Two of the three covariates of this model, histologic grade and FIGO stage, are established from the surgical specimen and are therefore not available at the time the preoperative blood sample is taken. The comparison reported here consequently asks whether the blood indices add information to a complete postoperative pathological assessment; it is not a test of preoperative risk stratification.

Discrimination was quantified using methods appropriate for right-censored data. Harrell’s concordance index was calculated with 95% confidence intervals, the difference in concordance between nested models was tested using the paired covariance of the two estimates, and inverse-probability-of-censoring-weighted time-dependent areas under the curve were estimated at 36 and 60 months. Optimism was quantified by bootstrap internal validation with 1000 resamples, from which optimism-corrected concordance indices and calibration slopes were obtained.

Model stability was examined further by refitting the model with Firth’s penalized likelihood. The proportional hazards assumption was tested using scaled Schoenfeld residuals for each covariate and globally; where a violation was detected the model was refitted with stratification on the offending variable. Linearity of continuous covariates on the log hazard scale was examined with penalized smoothing splines. The influence of individual observations on the NRBC% coefficient was assessed with DFBETA statistics scaled by the standard error of the coefficient, with deviance residuals, and with a leave-one-out procedure in which the model was refitted 142 times, each time omitting one patient. Two further sensitivity analyses refitted the model after excluding the single highest NRBC% observation and after excluding all values above 0.30%. Full diagnostic output is provided as Supplementary Material (Table S3). Given the zero-inflated distribution of NRBC%, a pre-specified sensitivity analysis modeled the variable as detectable (>0%) versus undetectable, and hazard ratios were additionally reported per standard deviation and per interquartile range, because a one-percentage-point increment exceeds the range observed in most patients.

Fourteen blood-derived indices were evaluated. No correction for multiple comparisons was applied to the primary analyses, which are exploratory; Benjamini–Hochberg false discovery rate adjusted *p*-values are reported alongside the unadjusted values. Spearman correlation coefficients between the indices were calculated to characterize their collinearity structure and are provided as Supplementary Material.

## 3. Results

### 3.1. Patient Characteristics

Of one hundred and ninety-five patients who underwent surgery for endometrioid-type endometrial cancer between 2018 and 2024, fifty-three were excluded: eighteen for coexistent adenomyosis, fifteen for uterine leiomyoma, six for active inflammation or infection, eight for missing preoperative complete blood count or incomplete records, and six for other reasons including hematological disease and prior chemotherapy or radiotherapy (Figure 1). A total of 142 patients were included in the analysis. The mean age was 59.75 ± 9.01 years. Patient demographics showed that 95 patients (66.9%) were younger than 65 years, while 47 patients (33.1%) were 65 years or older. The median gravidity was 2.0 (range: 0–11), and median parity was 2.0 (range: 0–11).

Regarding surgical approach, 93 patients (65.5%) underwent laparotomy while 49 patients (34.5%) had laparoscopic surgery. Lymph-node dissection patterns varied based on risk factors, with 25 patients (17.6%) not undergoing lymph-node dissection, 53 patients (37.3%) having only pelvic lymphadenectomy, and 64 patients (45.1%) receiving both pelvic and para-aortic lymphadenectomy.

Comorbidity analysis revealed that 53 patients (37.3%) had diabetes mellitus (DM), 65 patients (45.8%) had hypertension (HT), 34 patients (23.9%) had both diabetes and HT, 13 patients (9.2%) had CAD, and 59 patients (41.5%) had other additional diseases. These comorbidities were considered in the analysis as potential confounders of inflammatory-marker interpretation.

Histopathological examination showed that the majority of tumors were well-differentiated, with 104 patients (73.2%) having grade 1 tumors, 26 patients (18.3%) having grade 2 tumors, and 12 patients (8.5%) having grade 3 tumors. Tumor size analysis revealed that 27 patients (19%) had tumors smaller than 2 cm, while 115 patients (81%) had tumors 2 cm or larger.

Myometrial invasion assessment showed that 95 patients (66.9%) had invasion less than 50% of myometrial thickness, while 47 patients (33.1%) had invasion of 50% or greater. LVSI was present in 19 patients (13.4%) and stromal invasion was detected in 20 patients (14.1%).

Assessment of extrauterine spread showed serosa and adnexal involvement in four patients (2.8%), vaginal–parametrial involvement in two patients (1.4%), and distant metastasis in six patients (4.2%). Nodal status was classified on histopathology. Pelvic lymph nodes were surgically assessed in 117 patients (82.4%): 105 (73.9% of the cohort) were pN0 and 12 (8.5%) were pN1, while 25 patients (17.6%) were pNX. Para-aortic lymph nodes were surgically assessed in sixty-four patients (45.1%): sixty (42.3%) were pN0 and four (2.8%) were pN1, while seventy-eight patients (54.9%) were pNX. In univariate Cox regression, neither pelvic (*p* = 0.415) nor para-aortic nodal status (*p* = 0.105) was associated with overall survival at the variable level. Within the para-aortic variable the pNX category showed a lower hazard than pN0 (HR 0.48, 95% CI 0.24–0.96, *p* = 0.039); since para-aortic lymphadenectomy was performed for grade 3 tumors and for selected cases on the basis of intraoperative findings, the dissected group was by construction at higher risk, and this inverse association is interpreted as confounding by indication rather than as a protective effect (Table 1).

### 3.2. Preoperative Laboratory Parameters and Derived Indices

Preoperative laboratory parameters and derived indices are summarized in Table 2. The median values of key inflammatory markers were as follows: HB 12.90 g/dL (range: 8.10–16.70), hematocrit (HCT) 39.0% (range: 24.0–50.0), WBC count 8.02 × 10^3^/μL (range: 2.77–15.91), lymphocyte count 2.19 × 10^3^/μL (range: 0.74–6.78), monocyte count 0.54 × 10^3^/μL (range: 0.17–1.12), neutrophil count 4.99 × 10^3^/μL (range: 0.92–11.05), and PLT 286.50 × 10^3^/μL (range: 84.0–892.0).

Advanced hematological parameters showed specific patterns associated with prognosis. The NRBC% had a median of 0.0% (range: 0.0–2.50) and showed significant prognostic value (HR: 2.57, 95% CI: 1.14–5.79, *p* = 0.023). The IG% had a median of 0.30% (range: 0.0–1.60) and showed a non-significant trend toward association with survival (HR: 2.80, 95% CI: 0.92–8.52, *p* = 0.070). Preoperative APRI showed a median of 0.19 (range: 0.05–1.36) and showed a non-significant trend toward association with poor OS (HR: 5.19, 95% CI: 0.96–28.14, *p* = 0.057). Preoperative FIB-4 score with a median of 0.96 (range: 0.38–8.27) demonstrated significant prognostic value (HR: 1.41, 95% CI: 1.12–1.77, *p* = 0.004). Additionally, among the subset with available data (*n* = 76, 53.5%), the IBI showed a median of 12.17 (range: 0.00–76.78) and did not reach statistical significance (HR: 1.02, 95% CI: 0.996–1.050, *p* = 0.098) (Table 2).

### 3.3. Survival Outcomes

The median observed follow-up, calculated from the date of surgery to death or to the administrative censoring date of 1 June 2026, was 52.5 months (interquartile range: 38.5–64.0 months; range: 1–80 months). Since the median OS was not reached due to the high proportion of censored patients at the end of follow-up, the mean OS time was reported: 66.4 months (SE: 2.08; 95% CI: 62.3–70.5 months). Cumulative survival rates were 95.1% (95% CI: 89.9–97.6%) at 12 months, 88.0% (95% CI: 81.4–92.4%) at 24 months, 82.2% (95% CI: 74.8–87.6%) at 36 months, 76.4% (95% CI: 68.3–82.8%) at 48 months, and 74.1% (95% CI: 65.5–80.9%) at 60 months.

Of the 142 patients in the study cohort, 108 patients (76.1%) remained alive at the end of follow-up, while 34 patients (23.9%) had died. Age analysis revealed significant survival differences, with patients aged 65 years or older showing significantly worse outcomes compared to younger patients (HR: 2.20, 95% CI: 1.12–4.32, *p* = 0.021).

### 3.4. Univariate Analysis

Traditional preoperative inflammatory ratios and indices were analyzed as continuous variables. In the univariate analysis, the SII (*p* = 0.893), PIV (*p* = 0.436), LMR (*p* = 0.579), NLR (*p* = 0.263), PLR (*p* = 0.695), ICPI (*p* = 0.787), and GLR (*p* = 0.375) did not reach statistical significance. Preoperative SIRI demonstrated a borderline trend toward survival significance (*p* = 0.051), while preoperative MLR reached statistical significance (HR: 26.76, 95% CI: 2.15–332.49, *p* = 0.011). Calculated with the original binary scoring, ICPI had a median of 2.90 (range 0.00 to 8.50) and was unrelated to survival both as a continuous score (HR 1.02, 95% CI 0.90 to 1.15, *p* = 0.787) and when dichotomized at the published threshold of 2.9, which classified 61 patients (43.0%) as high (HR 1.42, 95% CI 0.73 to 2.79, *p* = 0.303; log-rank *p* = 0.303).

In univariate analysis, thirteen variables met the *p* < 0.10 threshold for multivariable candidacy: age (continuous and categorical), surgical approach, extent of lymphadenectomy, CAD, histologic grade, distant metastasis, vaginal or parametrial metastasis, NRBC (%), IG (%), SIRI, MLR, APRI, and FIB-4. As detailed in Section 2 (Statistical Analysis), surgical approach, extent of lymphadenectomy, and vaginal or parametrial metastasis were excluded from multivariable candidacy due to confounding by indication and small-cell instability, respectively, and one representative marker from each pair of collinear indices (FIB-4 over APRI; MLR over SIRI) was retained. The resulting candidate set of eight variables was entered simultaneously into the multivariable Cox regression model (Enter method).

### 3.5. Multivariable Analysis

In the multivariable Cox regression model (Table 3), histologic grade and NRBC (%) were the only variables that remained significantly associated with OS after simultaneous adjustment for all eight candidate variables.

**Figure 2:**
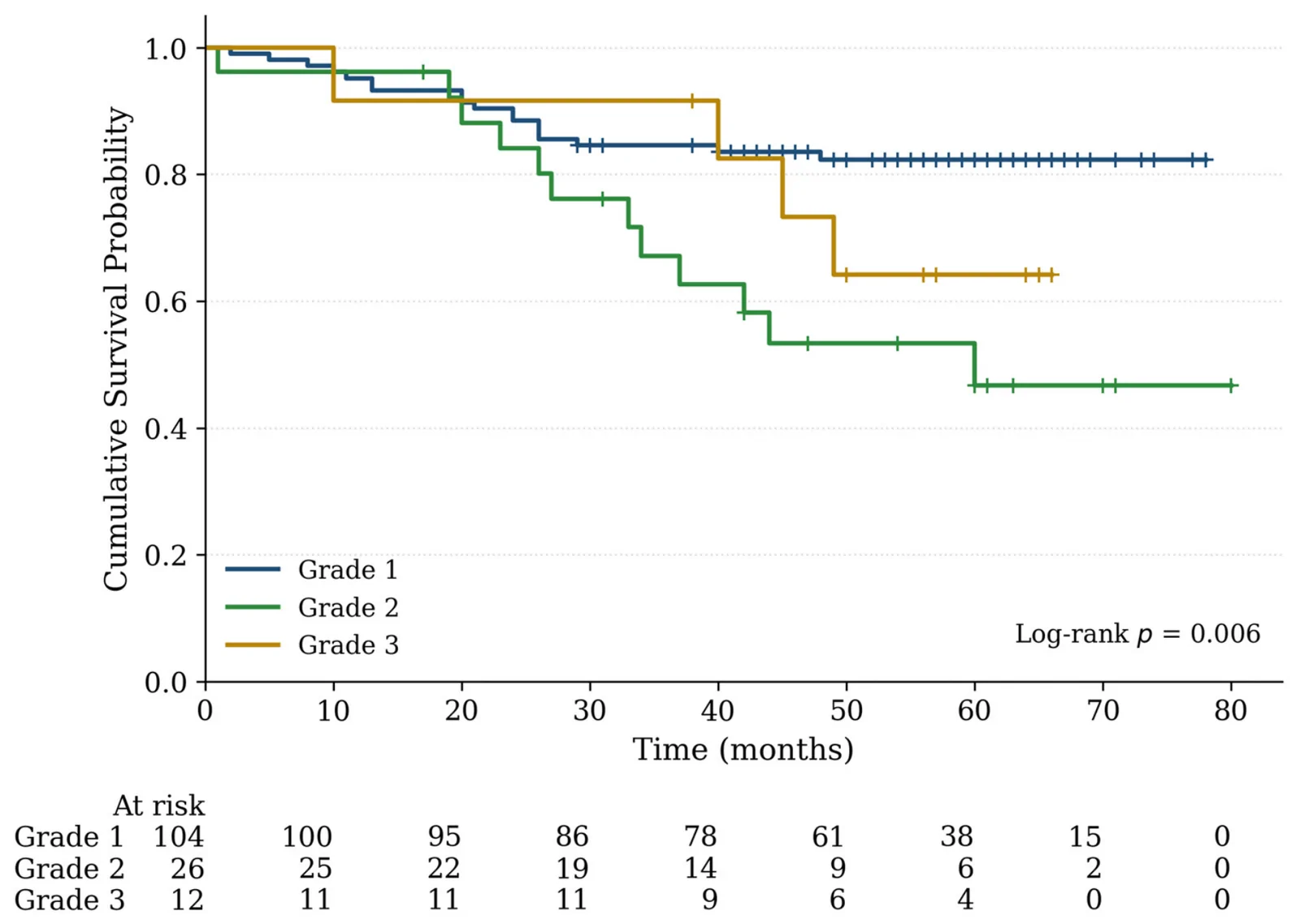
Kaplan–Meier survival curves stratified by histologic grade. Overall survival differed significantly across the three grade groups (log-rank *p* = 0.006); grade 2 patients showed the lowest observed survival, followed by grade 3 and grade 1. Tick marks indicate censored observations; the number of patients at risk at each time point is shown below the plot.

Histologic grade was significantly associated with OS overall (Wald = 9.513, df = 2, *p* = 0.009). Relative to grade 1, grade 2 tumors carried a higher hazard (HR: 3.50, 95% CI: 1.57–7.78, *p* = 0.002; Figure 2), whereas the estimate for grade 3 tumors was also elevated but did not reach significance and was imprecise (HR: 1.89, 95% CI: 0.57–6.30, *p* = 0.299). The grade 3 subgroup comprised only twelve patients with four events, and the confidence intervals for the two contrasts overlap substantially; these estimates should therefore not be read as indicating that grade 2 disease carries a worse prognosis than grade 3 disease.

Higher NRBC (%) remained independently associated with OS after adjustment, each 1% increase in the preoperative NRBC proportion corresponding to an approximately 3.3-fold higher hazard of death (HR: 3.30, 95% CI: 1.09–9.97, *p* = 0.034).

CAD (HR: 1.76, 95% CI: 0.59–5.22, *p* = 0.311), distant metastasis (HR: 1.31, 95% CI: 0.33–5.15, *p* = 0.697), age category (HR: 1.34, 95% CI: 0.61–2.92, *p* = 0.471), IG% (HR: 1.76, 95% CI: 0.44–7.14, *p* = 0.428), and FIB-4 (HR: 1.23, 95% CI: 0.93–1.63, *p* = 0.152) did not maintain independent statistical significance after multivariable adjustment, despite univariate association with OS. Preoperative MLR showed a similarly elevated but highly imprecise point estimate (HR: 16.08, 95% CI: 0.75–342.94, *p* = 0.075) that did not reach statistical significance; given the extremely wide confidence interval, this estimate should not be interpreted as a reliable effect size.

In the sensitivity analysis restricted to the complete-case subset with available CRP data (*n* = 76, 16 events; Table 4), IBI showed a non-significant trend toward an independent association with OS after adjustment for age category and grade (HR: 1.03, 95% CI: 1.00–1.05, *p* = 0.065); the overall model was not statistically significant (omnibus χ^2^ = 7.731, df = 4, *p* = 0.102).

### 3.6. Model Stability, Incremental Prognostic Value, and Discrimination

The pre-specified clinical model comprising age category, histologic grade, and FIGO stage yielded an events-per-variable ratio of 8.5. Within this model, age ≥ 65 years (HR 2.23, 95% CI 1.11–4.48, *p* = 0.025) and grade 2 relative to grade 1 (HR 3.16, 95% CI 1.46–6.82, *p* = 0.003) were associated with overall survival, whereas grade 3 (HR 2.01, 95% CI 0.60–6.75, *p* = 0.262) and FIGO stage ≥ II (HR 0.91, 95% CI 0.38–2.17, *p* = 0.835) were not.

Adding NRBC% (EPV 6.8) improved model fit: the likelihood-ratio test was significant (χ^2^ = 4.25, df = 1, *p* = 0.039), and NRBC% remained associated with overall survival after adjustment (HR 3.42 per 1% increase, 95% CI 1.46–8.04, *p* = 0.005; HR 1.33 per standard deviation, 95% CI 1.09–1.61; HR 1.13 per interquartile range, 95% CI 1.04–1.23). The estimate was robust to Firth’s penalized likelihood (HR 3.91, 95% CI 1.52–7.30, *p* = 0.011) and to stratification on histologic grade (HR 3.00, 95% CI 1.28–7.01, *p* = 0.011). Adding all four biomarkers likewise improved fit (χ^2^ = 11.63, df = 4, *p* = 0.020), although the events-per-variable ratio fell to 4.3 and this model should be regarded as exploratory (Table 5).

Improvement in model fit was not accompanied by improvement in discrimination. Harrell’s concordance index was 0.673 (95% CI 0.590–0.757) for the clinical model and 0.679 (95% CI 0.587–0.771) after the addition of NRBC%, a difference of 0.006 (95% CI −0.041 to 0.053, *p* = 0.814). Bootstrap internal validation with 1000 resamples gave optimism-corrected concordance indices of 0.638 and 0.640 respectively, with calibration slopes of 0.81 and 0.74. Time-dependent areas under the curve were 67.8% versus 69.1% at 36 months (*p* = 0.812) and 69.9% versus 70.3% at 60 months (*p* = 0.966).

The proportional hazards assumption held globally in the pre-specified model (*p* = 0.075) and for NRBC% (*p* = 0.290) but was violated for histologic grade (*p* = 0.041); refitting with stratification on grade resolved this (global *p* = 0.280) and did not materially alter the NRBC% estimate. In the exploratory eight-variable model the global test was also violated (*p* = 0.044), and stratification on grade again resolved it (global *p* = 0.097). NRBC% was linear on the log hazard scale (test for non-linearity *p* = 0.958).

NRBC% was detectable in 42 of 142 patients (29.6%), comprising thirty-three of one hundred and eight survivors (30.6%) and nine of thirty-four non-survivors (26.5%). Among patients with detectable values the median was 0.10% (interquartile range 0.10–0.20; maximum 2.50), so that even the highest observations were small in absolute terms. When modeled as detectable versus undetectable, NRBC carried no prognostic information (adjusted HR 0.79, 95% CI 0.36–1.71, *p* = 0.543; five-year overall survival 74.6% versus 73.4%, log-rank *p* = 0.700). Mortality by NRBC% band was 25.0% at 0%, 14.8% above 0 to 0.10%, 20.0% above 0.10 to 0.30%, 50.0% above 0.30 to 0.60% and 100% above 0.60%; only five patients had values exceeding 0.30%, of whom three died. The continuous association therefore rests on a small number of patients at the upper end of the distribution.

Diagnostics for individual observations gave a more precise picture of what supports this estimate; they are reported in full in Table S3. No single patient was influential in the conventional sense: scaled DFBETA values for the NRBC% coefficient ranged from −0.15 to 0.15, so that omitting any one observation moved the coefficient by at most one seventh of its standard error. Seven patients had deviance residuals beyond ±2, and all seven were patients with undetectable NRBC% who died within 24 months, that is, they are deaths that the model does not explain rather than patients with extreme marker values. In the leave-one-out procedure the hazard ratio ranged from 2.14 to 3.66 and the *p*-value from 0.002 to 0.655, and significance was lost in only one of the 142 refits. That single instance is decisive; however, it was the omission of the one patient with an NRBC% of 2.50 who died, after which the estimate became uninformative rather than null (HR 2.14, 95% CI 0.08 to 60.40, *p* = 0.655). Excluding all five patients with values above 0.30% left no interpretable estimate at all (HR 0.03, 95% CI 0.00 to 30.22, *p* = 0.324). The per-unit association is therefore not the product of a single aberrant point, but the range over which it is estimated is created almost entirely by the few patients at the top of the distribution, and once they are removed the data contain too little variation in NRBC% to support any estimate.

After Benjamini–Hochberg adjustment across the fourteen blood-derived indices evaluated, no index retained significance at the 0.05 level (FIB-4 adjusted *p* = 0.056; MLR 0.077; NRBC% 0.107). Spearman correlations documented the collinearity structure underlying marker selection (FIB-4 with APRI rho = 0.75; MLR with SIRI rho = 0.74; SII with PIV rho = 0.87; LMR with MLR rho = −1.00); ICPI, calculated with the original binary scoring, correlated only moderately with PLR (rho = 0.50), whereas the same formula applied to raw component values reproduced PLR almost exactly (rho = 0.999). By contrast, NRBC% and IG% correlated only weakly with every ratio-based index (|rho| ≤ 0.26), indicating that they carry information distinct from the conventional inflammatory ratios.

Patients with and without available C-reactive protein measurements did not differ significantly in age (*p* = 0.385), duration of follow-up (*p* = 0.737), NRBC% (*p* = 0.610), MLR (*p* = 0.572), FIB-4 (*p* = 0.080), vital status (*p* = 0.503), age category (*p* = 0.815), histologic grade (*p* = 0.097), or FIGO stage (*p* = 1.000), suggesting that the availability of C-reactive protein was unrelated to outcome.

Recurrence was recorded in four patients. In a supportive analysis using disease-free survival (38 events), NRBC% remained associated with outcome (HR 3.44, 95% CI 1.47–8.06, *p* = 0.004). Because recurrences were few, disease-free survival was largely driven by deaths, and this analysis should be regarded as a consistency check rather than as an independent endpoint.

## 4. Discussion

In this single-center cohort of 142 patients with endometrioid-type endometrial cancer, the composite inflammatory indices that dominate the current literature were not associated with OS when they were modeled as continuous variables. The SII, PIV, NLR, PLR, LMR, ICPI, and GLR were all non-significant. Among the conventional ratios, only MLR reached significance in univariate analysis, with the SIRI at the margin of significance. After simultaneous adjustment, histologic grade and the preoperative NRBC% were the only variables that remained significantly associated with survival, and a pre-specified model comparison showed that NRBC% improved model fit without improving discrimination. Because this pattern diverges from several widely cited series, a detailed comparison with those cohorts is warranted.

Matsubara et al. reported the prognostic value of pre-treatment SII in 442 patients with endometrial cancer and identified ROC-derived cut-off values of 931 for progression-free survival and 910 for OS [9]. An elevated SII was present in 30.5% of their patients and remained independently associated with both progression-free survival (HR 1.71, 95% CI 1.11 to 2.62, *p* = 0.014) and OS (HR 1.96, *p* = 0.011), and SII outperformed PLR in terms of discrimination for three-year OS (area under the curve [AUC] 0.709 versus 0.625, *p* = 0.008). Two features of that cohort are relevant to the discrepancy with our results. First, 21.7% of their patients had non-endometrioid histology, 34.2% were classified as grade 3 and 20.9% presented with FIGO stage III or IV disease, whereas our cohort was restricted to endometrioid carcinoma, 73.2% of tumors were grade 1 and 78.9% of patients had stage I disease. Second, the median SII in our series was 635.89 (range 143.11 to 2365.20), well below the cut-off values reported by Matsubara et al., so only a minority of our patients would have been assigned to the high-SII stratum. A marker whose prognostic signal is generated mainly by the upper tail of its distribution will lose power in a cohort in which that tail is sparsely populated.

The findings of Huang et al. offer a complementary explanation [10]. In 362 surgically treated patients, preoperative SII, NLR, PLR, and MLR were all associated with OS in univariate analysis, yet none of them survived multivariable adjustment; only lymphatic invasion (HR 21.35, 95% CI 4.78 to 95.41, *p* < 0.0001) and the postoperative SII measured three days after surgery (HR 8.74, 95% CI 1.45 to 51.65, *p* = 0.017) remained independent. The discriminative capacity of preoperative SII in that series was modest (AUC 0.645). Our results are therefore concordant with the multivariable rather than the univariate portion of that study: preoperative peripheral blood indices appear to encode information that is largely absorbed by pathological descriptors of tumor burden once these are entered into the same model. Huang et al. also argued that blood sampling after cytoreduction reflects the residual inflammatory state more faithfully than the preoperative sample. Our protocol used a single preoperative measurement obtained within seven days of surgery and did not include a postoperative time point, which limits any direct comparison on this specific question and identifies a concrete direction for future work in our population.

Cong et al. analyzed 1111 patients and dichotomized NLR, PLR, and MLR at ROC-derived thresholds of 2.14 (AUC 0.671), 131.82 (AUC 0.652), and 0.22 (AUC 0.630), respectively [28]. All three were independently associated with worse OS (NLR HR 2.71, 95% CI 1.83 to 4.02; PLR HR 2.75, 95% CI 1.90 to 3.97; MLR HR 1.72, 95% CI 1.20 to 2.45), and the combination of all three elevated ratios produced the strongest signal (HR 4.34, 95% CI 2.54 to 7.42), with a nomogram concordance index of 0.847. The absolute values in our cohort were closely comparable to those thresholds: our median NLR was 2.40, our median PLR 130.08 and our median MLR 0.25. The distributions of these markers were therefore not appreciably different, which makes a purely biological explanation for the divergence unlikely. The more plausible sources of difference are statistical power and case mix. Cong et al. observed their events in a cohort almost eight times larger and one that included stromal sarcoma, clear cell, serous, mixed, and carcinosarcoma histologies, whereas our analysis was confined to 34 deaths among 142 patients with pure endometrioid tumors. It is also notable that MLR carried the smallest independent effect in their multivariable model and was nonetheless the only conventional ratio to reach significance in ours, which suggests that the monocyte compartment may be the most consistent of the three across differing cohort structures.

The two prospective studies by Ronsini et al. address a different endpoint and, read alongside our data, help clarify what these indices actually measure. In the first study, 161 patients undergoing surgery for endometrial cancer were analyzed with deep myometrial infiltration as the outcome; NLR and MLR were significantly associated with infiltration of 50% or more (NLR log (OR) 0.15, *p* = 0.040; MLR log (OR) 0.30, *p* = 0.008) while PLR was not (*p* = 0.40), and NLR retained significance after adjustment for grading, histotype, and microsatellite status (log (OR) 0.18, *p* = 0.031) [29]. In the second, multicentric study of 163 patients with FIGO 2023 stage I disease, NLR rose stepwise across absent, less than 50% and 50% or greater myometrial infiltration (2.07, 2.35 and 2.68; *p* = 0.033), with parallel gradients for MLR (0.20, 0.23, 0.26; *p* = 0.029) and PLR (119, 140, 146; *p* = 0.043); NLR was also higher in diffuse lymphovascular space invasion (LVSI) than in LVSI-negative cases (3.23 versus 2.17, *p* = 0.010), and myometrial infiltration showed the strongest correlation with the inflammatory indices on multivariable analysis (beta 0.07, 95% CI 0.01 to 0.13, *p* = 0.041) [30]. These results position NLR, MLR, and PLR primarily as surrogates of local tumor burden and invasive behavior rather than as direct determinants of death. That distinction matters for the interpretation of our cohort, because in our patients the very pathological features that these indices track were themselves not associated with OS: myometrial invasion of 50% or greater carried an HR of 1.52 (*p* = 0.230) and LVSI an HR of 0.84 (*p* = 0.749). A biomarker that functions as a proxy for a variable with no measurable survival effect in a given cohort cannot be expected to be associated with survival in that cohort. The convergence of the two Ronsini studies on MLR and NLR, and our own finding that MLR was the best-performing conventional ratio, is consistent with this interpretation.

A methodological difference between our analysis and most of the studies cited above concerns the treatment of the biomarkers as continuous rather than dichotomized variables. We deliberately avoided data-derived cut-off values because such thresholds are optimized within the cohort that generates them and inflate apparent effect sizes. The published thresholds are markedly heterogeneous: the NLR cut-off was 2.14 in Cong et al. [28] and 3.01 in Huang et al. [10], the PLR cut-off was 131.82 [28] versus 169.62 [10], and the MLR cut-off was 0.22 [28] versus 0.31 [10], while the SII cut-off values differ by orders of magnitude between series because of divergent unit conventions [9,10]. This dispersion is itself evidence that optimized thresholds do not transport across cohorts. Continuous modeling avoids that source of optimism, but it carries a corresponding cost: if the relationship between an index and mortality is non-monotonic or confined to an extreme stratum, a single continuous hazard term will fail to detect it. Part of the difference between our null results and the positive findings of the dichotomized analyses is therefore attributable to the estimand rather than to the biology. The construction of the composite indices deserves a related comment, because it bears on how their null results should be read. ICPI is defined in its source publication as a weighted sum of three binary indicators rather than of the raw ratios [24], and the distinction has substantive consequences. When the same weights are applied to raw component values, the PLR term contributes approximately 364 of a median index value of 382.32 and the index becomes numerically almost indistinguishable from a rescaled PLR (Spearman rho = 0.999 in this cohort), so that the two variables cannot provide independent evidence. Calculated as originally specified, ICPI ranged from 0 to 8.5 with a median of 2.90, correlated only moderately with PLR (rho = 0.50) and remained unrelated to survival, whether modeled as a continuous score (HR 1.02, 95% CI 0.90 to 1.15, *p* = 0.787) or dichotomized at the published threshold of 2.9 (HR 1.42, 95% CI 0.73 to 2.79, *p* = 0.303; five-year overall survival 76.2% versus 71.4%, log-rank *p* = 0.303). The null result for ICPI in this cohort is therefore a property of the index itself rather than an artifact of how it was computed. More generally, composite indices that share components with the ratios they are compared against should be checked for numerical redundancy before their agreement is treated as corroboration.

A further methodological feature of our study is the exclusion of patients with histologically confirmed adenomyosis or leiomyoma. Both conditions are associated with a measurable systemic inflammatory response and are common incidental findings in hysterectomy specimens, so their presence in an unselected cohort introduces an inflammatory signal that is unrelated to the malignancy. To our knowledge, none of the five comparator studies applied this exclusion. The principle is not confined to these two conditions. In a multicenter study of 53 women with endometriosis and 40 women without the disease, plasma annexin A2 concentrations were significantly higher in stage III to IV endometriosis (*p* = 0.03) [31], which shows that benign inflammatory gynecological disease can shift the profile of candidate circulating biomarkers independently of any malignancy. The same collaboration reported a complementary observation for poly(ADP-ribose) polymerase: plasma and peritoneal concentrations did not differ between women with and without endometriosis, yet plasma levels were significantly higher in women with a history of infertility (*p* = 0.04) [32]. Taken together, these data indicate that the circulating biomarker profile of a gynecological population is shaped not only by the presence of a benign disease but also by coexisting reproductive phenotypes. Our exclusions removed two histologically defined confounders, but reproductive and endocrine characteristics were not recorded, so residual confounding of this kind cannot be excluded. The resulting cohort is methodologically cleaner, but the removal of these patients also removes variance from the inflammatory indices, and the narrower distribution reduces the power of any test based on those indices. The magnitude of this trade-off can be estimated from external data. In a retrospective cohort of 543 women undergoing hysteroscopy for abnormal uterine bleeding, hemoglobin concentration and platelet count differed significantly across etiological groups defined by the FIGO PALM-COEIN system, including endometrial polyp, endometrial hyperplasia and myomatosis (Kruskal–Wallis *p* < 0.001 and *p* < 0.01 respectively), with hemoglobin below the reference range in 17.3% and thrombocytopenia in 4.0% of the cohort [20]. This matters directly here, because platelet count enters PLR, SII, PIV, APRI, and FIB-4 as a numerator or denominator, and hemoglobin bears on the marrow-stress interpretation of NRBC%. Excluding adenomyosis and leiomyoma therefore removes a quantifiable, not merely theoretical, source of variation in precisely the components from which our indices are built. A second cost of this design deserves equal emphasis. Adenomyosis and leiomyoma were excluded on final histopathology, which is information that does not exist the moment a preoperative blood index is measured. The cohort is therefore more homogeneous in etiological terms than the population in which such a marker would actually be applied, and our estimates describe the behavior of these indices in a setting that cannot be identified prospectively. A clinically deployable preoperative marker would have to perform in unselected endometrioid carcinoma, incidental benign uterine pathology included. Both considerations point the same way: our null results should be compared with series in which benign uterine pathology was not excluded only with these differences in mind, and they should not be read as an estimate of how these indices would perform in routine preoperative practice.

A related constraint applies to the comparator itself. The pre-specified clinical model against which the indices were tested comprises age together with histologic grade and FIGO stage, and both of the pathological variables are determined on the surgical specimen. The incremental value reported here is therefore incremental to a complete postoperative pathological assessment, which is the most demanding benchmark available but not the one a preoperative marker would face in practice. What our results establish is that these indices add no discriminatory information to postoperative pathology. They do not establish, and were not designed to establish, whether the same indices could contribute to preoperative risk stratification, where the competing information is confined to imaging, endometrial biopsy grade, and clinical variables. That question requires a model built exclusively from preoperatively available data and remains open.

The association between preoperative NRBC% and OS (HR 3.30 per 1% increase, 95% CI 1.09 to 9.97, *p* = 0.034) was the only association to persist after multivariable adjustment and is the finding least anticipated from the existing literature, which is precisely why it requires the most careful handling. Circulating nucleated erythrocytes are normally confined to the bone marrow in adults, and their appearance in peripheral blood signals disruption of the marrow–blood barrier and stress erythropoiesis driven by tissue hypoxia, marrow infiltration and cytokine release, including interleukin-3, interleukin-6, and erythropoietin. In critically ill medical populations, the detection of nucleated red blood cells is strongly and dose-dependently associated with mortality [33]. None of the five comparator studies evaluated this parameter, and we are not aware of previous reports of its prognostic value in endometrial cancer.

Several further analyses define the standing of this finding more precisely than the hazard ratio alone. The coefficient itself is stable: it survived Firth’s penalized likelihood, survived stratification for the proportional hazards violation of grade, was linear on the log hazard scale, behaved consistently when disease-free survival was substituted for overall survival, and was not driven by any individual observation, the largest scaled DFBETA reaching only 0.15. What is not stable is the basis on which it can be estimated. The whole of the usable range of NRBC% is generated by a handful of patients, and when the single highest value is removed the estimate does not fall towards the null but loses all precision (HR 2.14, 95% CI 0.08 to 60.40). An estimate that cannot be reproduced without one particular patient is not a finding on which clinical inference can rest, however well behaved its diagnostics. Adding it to a pre-specified clinical model improved fit (likelihood-ratio *p* = 0.039). Yet the same model showed no gain in discrimination, whether measured by the concordance index (ΔC 0.006, 95% CI −0.041 to 0.053, *p* = 0.814) or by time-dependent areas under the curve at 36 and 60 months. Improvement in likelihood without improvement in discrimination is a recognized pattern for markers whose informative range is confined to a small tail of the distribution, and it argues against any immediate clinical role.

Three further observations temper the finding. First, when NRBC was dichotomized at the detection limit the association disappeared entirely (adjusted HR 0.79, 95% CI 0.36 to 1.71, *p* = 0.543; log-rank *p* = 0.700), showing that the signal derives from the magnitude of NRBC% among the few patients with detectable values rather than from detectability itself. Second, only five patients had values above 0.30%, of whom three died, so the continuous estimate rests on a very small number of observations at the upper end of the distribution. Third, the values that carry the association are minimal in absolute terms. Nucleated erythrocytes are normally absent from adult peripheral blood, so a reading of 0.1% is qualitatively abnormal yet quantitatively negligible, and the clinical meaning of elevations of this magnitude has not been established in any solid tumor population. The marker therefore cannot be interpreted through a conventional abnormal-versus-normal framework, and the distinction between a genuine low-grade marrow signal and analytical noise at the limit of reporting resolution cannot be resolved with these data. After Benjamini–Hochberg adjustment across the fourteen indices was evaluated, NRBC% did not retain significance (adjusted *p* = 0.107), and neither did any other index. The finding should therefore be regarded as hypothesis-generating and requires confirmation in larger, prospectively collected cohorts before any clinical use is considered.

Among the hepatic and metabolic indices, FIB-4 was significantly associated with OS in univariate analysis (HR 1.41, 95% CI 1.12 to 1.77, *p* = 0.004) but lost significance after adjustment (HR 1.23, 95% CI 0.93 to 1.63, *p* = 0.152), and APRI showed only a trend (*p* = 0.057). FIB-4 incorporates age and platelet count in its formula, so part of its univariate signal is likely to re-express the effect of age, which was itself associated with OS (HR 2.20 for age 65 years or older, *p* = 0.021). External data support this interpretation: in a gynaecological population, platelet count, the other principal component of FIB-4, declines with advancing age (r = −0.22, *p* < 0.0001) [20], so both terms of the index move in the same direction as patients grow older. The inflammatory burden index, which combines C-reactive protein with the neutrophil-to-lymphocyte ratio [34], showed a consistent but non-significant trend in the complete-case subset with available C-reactive protein data (univariate HR 1.02, *p* = 0.098; adjusted HR 1.03, 95% CI 1.00 to 1.05, *p* = 0.065 in a model whose omnibus test was not significant). Because C-reactive protein was available in only 76 of 142 patients (53.5%), this subanalysis is underpowered and cannot resolve whether the addition of an acute-phase reactant improves on cell-count-based indices in this setting. Coronary artery disease was associated with OS in univariate analysis (HR 2.76, 95% CI 1.14 to 6.69, *p* = 0.025) but not after adjustment (HR 1.76, *p* = 0.311). Since overall survival counts deaths from any cause, part of this univariate association plausibly reflects competing non-cancer mortality rather than an effect on tumor behavior, and a competing-risks framework with cause-specific endpoints would be needed to separate the two.

An incidental finding reinforces the interpretation offered above. In the pre-specified clinical model, FIGO stage carried no prognostic information in this cohort (HR 0.91, 95% CI 0.38 to 2.17, *p* = 0.835), and histologic grade accounted for essentially all of the pathological signal. Together with the null results for myometrial invasion (HR 1.52, *p* = 0.230) and LVSI (HR 0.84, *p* = 0.749), this indicates that in a cohort of this composition the anatomical descriptors of tumor burden are themselves only weakly related to overall survival. A peripheral blood index that behaves as a surrogate for tumor burden, as the studies of Ronsini et al. suggest these indices do, therefore has little opportunity to demonstrate prognostic value here, and this offers a coherent explanation for the null results observed across the conventional inflammatory ratios.

Two features of the cohort require comment. Five-year overall survival was 74.1% although 78.9% of patients had stage I disease and 73.2% had grade 1 tumors, which is lower than stage and grade alone would suggest. Overall survival counts deaths from any cause in a population whose mean age was 59.8 years and in which 33.1% of patients were 65 years or older, with diabetes, hypertension, and coronary artery disease all well represented, and the univariate association between coronary artery disease and survival that did not persist after adjustment points in the same direction. This interpretation could not be confirmed, because the cause of death was not recorded for patients who died outside our institution and cancer-specific survival could not be constructed. The same limitation of the records applies to adjuvant treatment: therapy was recommended in accordance with prevailing guidelines, but a proportion of patients received it elsewhere or did not attend, and the treatment actually delivered could not be verified. The associations reported here therefore describe all-cause mortality in a treated cohort rather than the isolated effect of these indices on tumor behavior.

The behavior of histologic grade in our model requires comment. Grade was significantly associated with survival overall (Wald 9.513, *p* = 0.009), but the effect was driven by grade 2 tumors (HR 3.50, 95% CI 1.57 to 7.78, *p* = 0.002) while grade 3 tumors did not differ significantly from grade 1 (HR 1.89, 95% CI 0.57 to 6.30, *p* = 0.299). This ordering is biologically implausible and is best explained by the small size of the grade 3 subgroup (twelve patients, four events), which yields a wide and unstable estimate, together with the well-documented interobserver variability of the grade 2 category and possible differences in adjuvant treatment allocation between grade strata. The result should be read as evidence that grade carries prognostic information in this cohort, not as evidence that grade 2 disease is more lethal than grade 3 disease.

Several limitations constrain the interpretation of these findings. The study is retrospective and single-center, and treatment allocation was not randomized. The number of events (34) relative to the number of model parameters produced an events-per-variable ratio below the conventionally recommended range, so all multivariable estimates are exploratory. Molecular classification according to current European guidance was not available for this cohort, which is a substantial omission given that the molecular subgroups now carry prognostic weight that may confound or modify the associations reported here [3]. Adjuvant treatment could not be modeled as a covariate, because the therapy actually delivered could not be verified for the substantial proportion of patients treated at other institutions. Since adjuvant therapy is allocated on the basis of the same pathological variables that enter the model, residual confounding by treatment cannot be excluded, and it is one plausible contributor to the ordering of the grade estimates. C-reactive protein and CA125 were available in only about half of the patients. Blood sampling was performed at a single preoperative time point, so the dynamic behavior of these indices after surgery, which Huang et al. found to be the more informative signal [10], could not be assessed. Nodal status could not be established pathologically in all patients: pelvic nodes were not assessed in 25 patients (17.6%) and para-aortic nodes in 78 patients (54.9%). These cases are reported as pNX and were not merged with pathologically node-negative cases, but occult nodal disease cannot be excluded in this subgroup, and the FIGO stage assigned to these patients rests on uterine factors alone and should be read as a minimum stage. Because lymphadenectomy was omitted precisely in patients judged preoperatively and intraoperatively to be at low risk, pNX status is confounded by indication and cannot be interpreted as a biological variable. The proportional hazards assumption was violated globally in the exploratory eight-variable model (*p* = 0.044) and for histologic grade in the pre-specified model (*p* = 0.041); stratification on grade resolved both, but the unstratified estimates should be read with this in mind. Fourteen indices were evaluated without correction for multiple comparisons in the primary analyses, and after Benjamini–Hochberg adjustment no index retained significance, so chance findings cannot be excluded. Staging followed the FIGO 2009 system and the cohort was not restaged according to the FIGO 2023 revision, because molecular classification was unavailable and LVSI was not quantified as focal or substantial; our stage assignments are therefore not directly comparable with cohorts staged under FIGO 2023. Only four recurrences were recorded, so the disease-free survival analysis was dominated by deaths and cannot be regarded as an independent endpoint. Finally, overall survival was the principal endpoint and counts deaths from any cause. The cause of death was not recorded for patients who died outside our institution, so deaths could not be classified as cancer-related or non-cancer and cancer-specific survival could not be analyzed; a competing-risks framework would be required to separate the two, and this constraint applies to every association reported here. The exclusion of adenomyosis and leiomyoma was applied on final histopathology and therefore defines a cohort that cannot be identified before surgery; a sensitivity analysis including all endometrioid carcinomas irrespective of incidental benign uterine pathology would have quantified the effect of this design choice, but the records of the excluded patients were not retained in the analysis dataset and could not be recovered, so this analysis could not be performed. The clinical model against which the indices were compared contains histologic grade and FIGO stage, both obtained from the surgical specimen, so the study does not demonstrate the usefulness or otherwise of these markers for genuinely preoperative stratification. No external validation cohort was available. Taken together, these constraints mean that every multivariable estimate in this study, including that for NRBC%, should be regarded as exploratory and as requiring external validation before it informs practice.

The null results reported here should not be read as evidence that the conventional inflammatory ratios lack prognostic relevance in endometrial cancer generally. With 142 patients and 34 events, the study had limited power to detect modest effects, and the confidence intervals around several estimates are wide enough to accommodate clinically meaningful associations. What the data support is a narrower and more defensible claim: in a histologically homogeneous endometrioid population enriched for stage I disease and from which benign uterine pathology has been excluded, these indices did not add prognostic information beyond conventional pathological variables, and this was demonstrated by formal model comparison rather than inferred from a series of non-significant *p*-values.

The strengths of the study lie in its design. The cohort is histologically homogeneous, restricted to endometrioid carcinoma and additionally purified by the exclusion of adenomyosis and leiomyoma on final pathology, which removes two frequent sources of inflammatory confounding that were not controlled in the comparator series [9,10,28,29,30]. Twelve inflammatory and metabolic indices were evaluated head-to-head in the same patients using identical modeling assumptions, which permits a direct comparison of their relative performance rather than the selective reporting of a single favorable marker. Taken together with the published evidence, our results suggest that the prognostic utility of peripheral blood inflammatory indices in endometrial cancer is conditional on cohort composition and is most evident when advanced-stage and non-endometrioid cases are included, and that in a histologically homogeneous endometrioid population enriched for stage I disease these indices add little beyond conventional pathological variables. Within such a population, markers of marrow stress such as NRBC% may prove more informative than ratio-based inflammatory indices, a possibility that now needs prospective testing.

## 5. Conclusions

In this homogeneous cohort of 142 patients with endometrioid-type endometrial cancer, none of the fourteen preoperative blood-derived indices evaluated, spanning inflammatory and immune-cell, hematological and metabolic categories, were associated with overall survival after adjustment, with the single exception of NRBC%. In univariate analysis three indices reached nominal significance, MLR (*p* = 0.011), FIB-4 (*p* = 0.004), and NRBC% (*p* = 0.023); of these, only NRBC% retained nominal significance in the multivariable model, and none survived correction for multiple comparisons. Histologic grade was the only clinicopathological variable that remained significantly associated with survival.

The question posed at the outset was whether these indices add prognostic information beyond established clinicopathological variables, assessed against a model that includes grade and FIGO stage as determined on the surgical specimen. Formal model comparison answers it directly: adding NRBC% to a pre-specified clinical model improved model fit but produced no measurable gain in discrimination, and the same was true of the full biomarker panel. No index retained significance after correction for multiple comparisons, and the association of NRBC% disappeared when the variable was dichotomized at the detection limit.

These findings indicate that the prognostic behavior of blood-derived indices in endometrial cancer is conditional on cohort composition. In series enriched for advanced-stage and non-endometrioid disease they behave as markers of tumor burden and are associated with survival; in a purely endometrioid population enriched for stage I disease and from which adenomyosis and leiomyoma have been excluded, and in which FIGO stage itself carried no prognostic signal, they add little beyond conventional pathological variables.

Given the number of events, the events-per-variable ratio and the absence of external validation, every multivariable finding reported here is exploratory and hypothesis-generating. Markers of marrow stress such as NRBC% warrant dedicated evaluation in larger prospective cohorts with integrated molecular classification, serial preoperative and postoperative sampling and adjudicated cause of death, but none of the parameters studied here are ready to inform clinical decision-making.

## Figures and Tables

**Table 1:** Patient demographics, clinical characteristics, and survival analysis.

Variable	Category	Mean ± SD/Median (Min–Max)	Total	Survival Status		*p*	HR (95% CI)
				Alive	Deceased		
Age (Year)		59.75 ± 9.01	142 (100%)	58.99 ± 8.88	62.15 ± 9.16	0.084	1.03 (0.99–1.07)
	<65	54.79 ± 6.10	95 (66.9%)	78 (82.1%)	17 (17.9%)	**0.021**	1.00 (Ref)
	≥65	69.77 ± 4.44	47 (33.1%)	30 (63.8%)	17 (36.2%)		2.20 (1.12–4.32)
Gravity		2.0 (0–11)	142 (100%)	2.0 (0–11)	2.0 (0–8)	0.213	0.89 (0.74–1.07)
Parity		2.0 (0–11)	142 (100%)	2.0 (0–11)	2.0 (0–8)	0.364	0.91 (0.74–1.11)
Operation	Laparotomy		93 (65.5%)	62 (66.7%)	31 (33.3%)	**0.** **004**	5.78 (1.77–18.91)
	Laparoscopy		49 (34.5%)	46 (93.9%)	3 (6.1%)		1.00 (Ref)
Lymph-Node Dissection						0.088	
	Not Done		25 (17.6%)	20 (80.0%)	5 (20.0%)		1.00 (Ref)
	Only Pelvic		53 (37.3%)	45 (84.9%)	8 (15.1%)	0.570	0.72 (0.24–2.21)
	Pelvic and Para-aortic		64 (45.1%)	43 (67.2%)	21 (32.8%)	0.292	1.69 (0.64–4.48)
DM	Yes		53 (37.3%)	41 (77.4%)	12 (22.6%)	0.786	0.91 (0.45–1.83)
	No		89 (62.7%)	67 (75.3%)	22 (24.7%)		1.00 (Ref)
HT	Yes		65 (45.8%)	48 (73.8%)	17 (26.2%)	0.637	1.18 (0.60–2.30)
	No		77 (54.2%)	60 (77.9%)	17 (22.1%)		1.00 (Ref)
DM+HT	Yes		34 (23.9%)	24 (70.6%)	10 (29.4%)	0.406	1.37 (0.65–2.86)
	No		108 (76.1%)	84 (77.8%)	24 (22.2%)		1.00 (Ref)
CAD	Yes		13 (9.2%)	7 (53.8%)	6 (46.2%)	**0.** **025**	2.76 (1.14–6.69)
	No		129 (90.8%)	101 (78.3%)	28 (21.7%)		1.00 (Ref)
Other Disease	Yes		59 (41.5%)	41 (69.5%)	18 (30.5%)	0.102	1.76 (0.89–3.44)
	No		83 (58.5%)	67 (80.7%)	16 (19.3%)		1.00 (Ref)
Grade						**0.014**	
	Grade 1		104 (73.2%)	86 (82.7%)	18 (17.3%)		1.00 (Ref)
	Grade 2		26 (18.3%)	14 (53.8%)	12 (46.2%)	**0.002**	3.11 (1.50–6.47)
	Grade 3		12 (8.5%)	8 (66.7%)	4 (33.3%)	0.258	1.87 (0.63–5.52)
Tumor Size (cm)		3.20 (0.30–9.50)	142 (100%)	3.10 (0.30–9.50)	3.75 (0.70–8.00)	0.739	1.03 (0.87–1.22)
	<2 cm	1.00 (0.30–1.90)	27 (19%)	22 (81.5%)	5 (18.5%)	0.475	1.00 (Ref)
	≥2 cm	4.00 (2.00–9.50)	115 (81%)	86 (74.8%)	29 (25.2%)		1.41 (0.55–3.65)
Myometrial Invasion	<50%		95 (66.9%)	75 (78.9%)	20 (21.1%)	0.230	1.00 (Ref)
	≥50%		47 (33.1%)	33 (70.2%)	14 (29.8%)		1.52 (0.77–3.01)
LVSI	Yes		19 (13.4%)	15 (78.9%)	4 (21.1%)	0.749	0.84 (0.30–2.40)
	No		123 (86.6%)	93 (75.6%)	30 (24.4%)		1.00 (Ref)
Stromal Invasion	Yes		20 (14.1%)	17 (85.0%)	3 (15.0%)	0.273	0.52 (0.16–1.69)
	No		122 (85.9%)	91 (74.6%)	31 (25.4%)		1.00 (Ref)
Pelvic Lymph-Node Status						0.412	
	pN0 (dissected, negative)		105 (73.9%)	81 (77.1%)	24 (22.9%)		1.00 (Ref)
	pN1 (dissected, positive)		12 (8.5%)	7 (58.3%)	5 (41.7%)	0.179	1.94 (0.74–5.09)
	pNX (not assessed)		25 (17.6%)	20 (80.0%)	5 (20.0%)	0.803	0.88 (0.34–2.32)
Para-aortic Lymph-Node Status						0.103	
	pN0 (dissected, negative)		60 (42.3%)	40 (66.7%)	20 (33.3%)		1.00 (Ref)
	pN1 (dissected, positive)		4 (2.8%)	3 (75.0%)	1 (25.0%)	0.992	0.99 (0.13–7.40)
	pNX (not assessed)		78 (54.9%)	65 (83.3%)	13 (16.7%)	0.039	0.48 (0.24–0.96)
Adnexal or Serosal Metastasis	Yes		4 (2.8%)	2 (50.0%)	2 (50.0%)	0.196	2.57 (0.62–10.73)
	No		138 (97.2%)	106 (76.8%)	32 (23.2%)		1.00 (Ref)
Vaginal or Parametrial Metastasis	Yes		2 (1.4%)	0 (0%)	2 (100%)	**0.** **042**	4.42 (1.05–18.54)
	No		140 (98.6%)	108 (77.1%)	32 (22.9%)		1.00 (Ref)
Distant Metastasis	Yes		6 (4.2%)	2 (33.3%)	4 (66.7%)	**0.** **022**	3.40 (1.19–9.71)
	No		136 (95.8%)	106 (77.9%)	30 (22.1%)		1.00 (Ref)
FIGO Stage	Stage 1		112 (78.9%)	88 (78.6%)	24 (21.4%)	0.234	1.00 (Ref)
	Stage ≥ 2		30 (21.1%)	20 (66.7%)	10 (33.3%)		1.57 (0.75–3.28)

Data are presented as mean ± standard deviation, median (minimum–maximum), or frequency (percentage). *p*-values are derived from univariate Cox proportional hazards regression analysis. Bold *p*-values indicate statistical significance (*p* < 0.05). SD: standard deviation; HR: hazard ratio; CI: confidence interval; Ref: reference; LND: lymph-node dissection; DM: diabetes mellitus; HT: hypertension; CAD: coronary artery disease; LVSI: ymphovascular space involvement. pN0: nodal region surgically dissected and histologically negative; pN1: histologically confirmed nodal metastasis; pNX: nodal region not surgically assessed. Patients who did not undergo lymphadenectomy were not classified as node-negative. No patient underwent para-aortic dissection without concurrent pelvic dissection. For variables with more than two categories the variable-level *p*-value is derived from the likelihood-ratio test; for binary and continuous variables *p*-values are derived from the Wald test.

**Table 2:** Preoperative laboratory parameters, hematologic indices, and survival analysis.

Variable	Category	Median (Min–Max)	Total	Survival Status		*p*	HR (95% CI)
				Alive	Deceased		
HB (g/dL)		12.90 (8.10–16.70)	142 (100%)	12.90 (8.30–16.00)	12.90 (8.10–16.70)	0.538	0.94 (0.77–1.15)
HCT (%)		39.0 (24.0–50.0)	142 (100%)	39.00 (24.00–50.00)	38.00 (27.00–48.00)	0.428	0.97 (0.90–1.04)
WBC (×10^3^/μL)		8.02 (2.77–15.91)	142 (100%)	7.81 (2.77–15.91)	8.48 (3.78–13.56)	0.838	0.99 (0.86–1.13)
LYMPH (×10^3^/μL)		2.19 (0.74–6.78)	142 (100%)	2.21 (0.75–6.10)	2.17 (0.74–6.78)	0.425	0.83 (0.52–1.31)
MONO (×10^3^/μL)		0.54 (0.17–1.12)	142 (100%)	0.54 (0.17–1.12)	0.58 (0.24–1.04)	0.569	1.75 (0.26–12.03)
NEUT (×10^3^/μL)		4.99 (0.92–11.05)	142 (100%)	5.00 (0.92–11.05)	4.94 (1.75–10.11)	0.966	1.00 (0.85–1.19)
PLT (×10^3^/μL)		286.50 (84.0–892.0)	142 (100%)	289.50 (163.00–530.00)	279.00 (84.00–892.00)	0.530	0.999 (0.994–1.003)
NRBC (%)		0.00 (0.00–2.50)	142 (100%)	0.00 (0.00–0.60)	0.00 (0.00–2.50)	**0.023**	2.57 (1.14–5.79)
IG%		0.30 (0.00–1.60)	142 (100%)	0.30 (0.00–1.50)	0.40 (0.00–1.60)	0.070	2.80 (0.92–8.52)
CRP (mg/L)		5.00 (0.00–34.25)	76 (53.5%)	4.43 (0.00–34.25)	6.43 (2.05–24.76)	0.208	1.04 (0.98–1.12)
CA125 (U/mL)		17.95 (4.70–274.80)	80 (56.3%)	15.65 (4.70–274.80)	19.35 (8.90–201.30)	0.580	1.00 (0.99–1.01)
SII		635.89 (143.11–2365.20)	142 (100%)	649.98 (198.11–2365.20)	624.98 (143.11–1481.88)	0.893	1.000 (0.999–1.001)
SIRI		1.24 (0.16–4.80)	142 (100%)	1.24 (0.16–3.80)	1.27 (0.24–4.80)	0.051	1.410 (0.998–2.003)
PIV		330.11 (62.97–1541.15)	142 (100%)	330.11 (65.11–1233.66)	327.32 (62.97–1541.15)	0.436	1.000 (0.999–1.002)
LMR		3.97 (1.37–16.54)	142 (100%)	4.06 (1.92–12.27)	3.87 (1.37–16.54)	0.579	0.94 (0.75–1.17)
MLR		0.25 (0.06–0.73)	142 (100%)	0.25 (0.08–0.52)	0.26 (0.06–0.73)	**0.** **011**	26.76 (2.15–332.49)
NLR		2.40 (0.58–9.73)	142 (100%)	2.34 (0.61–9.73)	2.60 (0.58–7.87)	0.263	1.13 (0.91–1.41)
PLR		130.08 (60.09–381.75)	142 (100%)	129.55 (60.09–381.75)	135.50 (76.69–239.16)	0.695	1.00 (0.99–1.01)
ICPI		2.90 (0.00–8.50)	142 (100%)	2.90 (0.00–8.50)	4.25 (0.00–8.50)	0.787	1.018 (0.897–1.154)
APRI		0.19 (0.05–1.36)	142 (100%)	0.19 (0.07–0.52)	0.18 (0.05–1.36)	0.057	5.19 (0.96–28.14)
FIB-4		0.96 (0.38–8.27)	142 (100%)	0.93 (0.39–2.75)	1.06 (0.38–8.27)	**0.004**	1.41 (1.12–1.77)
IBI		12.17 (0.00–76.78)	76 (53.5%)	11.40 (0.00–61.67)	18.26 (1.20–76.78)	0.098	1.020 (0.996–1.050)
GLR		51.92 (16.23–304.00)	142 (100%)	51.29 (16.23–304.00)	59.26 (18.29–244.42)	0.375	1.003 (0.996–1.010)

Data are presented as median (minimum–maximum) or frequency (percentage). *p*-values are derived from univariate Cox proportional hazards regression analysis. Bold *p*-values indicate statistical significance (*p* < 0.05). Within each row the hazard ratio and its confidence limits are reported to the same number of decimal places. Because fourteen blood-derived indices were evaluated, Benjamini–Hochberg false discovery rate adjusted *p*-values are as follows: FIB-4 0.056; MLR 0.077; NRBC% 0.107; SIRI 0.160; APRI 0.160; IG% 0.163; IBI 0.196; NLR 0.460; GLR 0.583; PIV 0.610; LMR 0.737; PLR 0.811; ICPI 0.848; SII 0.893. No index retained significance at the 0.05 level after adjustment. Hazard ratios are expressed per one unit of the variable concerned. For MLR (observed range 0.06 to 0.73) and NRBC% (observed range 0.00 to 2.50) a one-unit increment exceeds the range observed in the cohort, which is the principal reason for the width of the corresponding confidence intervals; hazard ratios rescaled per standard deviation and per interquartile range are reported for NRBC% in Section 3.6. APRI: aspartate transaminase-to-platelet ratio index; CA125: cancer antigen 125; CI: confidence interval; CRP: C-reactive protein; FIB-4: fibrosis index based on four factors; GLR: glucose-to-lymphocyte ratio; HB: hemoglobin; HCT: hematocrit; HR: hazard ratio; IBI: inflammatory burden index; ICPI: inflammation-combined prognostic index; IG: immature granulocyte; LMR: lymphocyte-to-monocyte ratio; LYMPH: lymphocyte count; MLR: monocyte-to-lymphocyte ratio; MONO: monocyte count; NEUT: neutrophil count; NLR: neutrophil-to-lymphocyte ratio; NRBC: nucleated red blood cell; PIV: pan-immune–inflammation value; PLR: platelet-to-lymphocyte ratio; PLT: platelet count; SII: systemic immune–inflammation index; SIRI: systemic inflammation response index; WBC: white blood cell count.

**Table 3:** Multivariable Cox proportional hazards regression analysis for overall survival.

Variable	B	SE	Wald	df	*p*-Value	HR (95% CI)
Age category (≥65 vs. <65)	0.289	0.400	0.521	1	0.471	1.34 (0.61–2.92)
CAD	0.563	0.556	1.027	1	0.311	1.76 (0.59–5.22)
Grade (Ref: Grade 1)			9.513	2	**0.009**	-
-Grade 2 vs. Grade 1	1.252	0.408	9.405	1	**0.002**	3.50 (1.57–7.78)
-Grade 3 vs. Grade 1	0.637	0.614	1.077	1	0.299	1.89 (0.57–6.30)
Distant Metastasis	0.271	0.698	0.151	1	0.697	1.31 (0.33–5.15)
NRBC (%)	1.194	0.564	4.488	1	**0.034**	3.30 (1.09–9.97)
IG (%)	0.566	0.714	0.629	1	0.428	1.76 (0.44–7.14)
MLR	2.777	1.561	3.164	1	0.075	16.08 (0.75–342.94)
FIB-4	0.206	0.144	2.050	1	0.152	1.23 (0.93–1.63)

B: regression coefficient; CAD: coronary artery disease; CI: confidence interval; df: degrees of freedom; FIB-4: fibrosis-4 index; HR: hazard ratio; IG: immature granulocyte; MLR: monocyte-to-lymphocyte ratio; NRBC: nucleated red blood cell; SE: standard error. Bold *p*-values indicate statistical significance (*p* < 0.05). All covariates were entered simultaneously into the model (Enter method) based on a pre-specified set of candidates that were clinically relevant and reached *p* < 0.10 in univariate analysis; no variables were removed through automated stepwise selection. Hazard ratios for the continuous variables are expressed per one unit; for MLR and NRBC% this increment is larger than the range observed in the cohort, and the corresponding confidence intervals should be read accordingly.

**Table 4:** Sensitivity analysis of the inflammatory burden index (IBI) in the complete-case subset (*n* = 76).

Variable	B	SE	Wald	df	*p*-Value	HR (95% CI)
Age category (≥65 vs. <65)	0.621	0.518	1.438	1	0.230	1.86 (0.67–5.14)
Grade (Ref: Grade 1)			3.188	2	0.203	-
-Grade 2 vs. Grade 1	0.301	0.790	0.145	1	0.703	1.35 (0.29–6.36)
-Grade 3 vs. Grade 1	1.078	0.604	3.186	1	0.074	2.94 (0.90–9.61)
IBI	0.026	0.014	3.398	1	0.065	1.03 (1.00–1.05)

B: regression coefficient; CI: confidence interval; df: degrees of freedom; HR: hazard ratio; IBI: inflammatory burden index; SE: standard error. Because C-reactive protein (and therefore IBI) was available for only 76 of 142 patients (53.5%), IBI was not included in the primary multivariable model (Table 3) to avoid a substantial reduction in sample size through listwise deletion. This table presents a sensitivity analysis restricted to the complete-case subset (*n* = 76, 16 events) in which IBI was entered simultaneously (Enter method) alongside age category and grade. With sixteen events and four model parameters, the events-per-variable ratio (4.0) falls below the range generally considered stable (5–9), and results should be interpreted as exploratory. The overall model was not statistically significant (omnibus likelihood ratio χ^2^ = 7.731, df = 4, *p* = 0.102).

**Table 5:** Pre-specified model comparison, incremental prognostic value, and discrimination.

	M0: Clinical	M1: Clinical + NRBC%	M2: Clinical + Full Panel
Covariates	age category, grade, FIGO stage	M0 + NRBC%	M1 + IG%, MLR, FIB-4
Parameters	4	5	8
Events per variable	8.5	6.8	4.3
Likelihood-ratio test vs. M0	—	χ^2^ = 4.25, df 1, *p* = 0.039	χ^2^ = 11.63, df 4, *p* = 0.020
Harrell’s C (95% CI)	0.673 (0.590–0.757)	0.679 (0.587–0.771)	0.724 (0.643–0.805)
ΔC vs. M0 (95% CI)	—	0.006 (−0.041 to 0.053), *p* = 0.814	—
Bootstrap-corrected C	0.638	0.640	—
Calibration slope	0.81	0.74	—
Time-dependent AUC, 36 months	67.8%	69.1% (*p* = 0.812)	—
Time-dependent AUC, 60 months	69.9%	70.3% (*p* = 0.966)	—
NRBC% hazard ratio	—	3.42 (1.46–8.04), *p* = 0.005	3.10 (1.04–9.22), *p* = 0.041

AUC: area under the curve; CI: confidence interval; NRBC: nucleated red blood cell. M0 was specified a priori on clinical grounds rather than on univariate *p*-values. Optimism-corrected concordance indices and calibration slopes were obtained by bootstrap internal validation with 1000 resamples. Time-dependent areas under the curve were estimated with inverse probability of censoring weighting. M2 is exploratory, its events-per-variable ratio falling below the conventionally recommended range.

## Data Availability

The data presented in this study are available on request from the corresponding author due to restrictions (e.g., privacy or ethical reasons).

## Supplementary Materials

The following supporting information can be downloaded at: https://www.mdpi.com/article/10.3390/medicina62091767/s1, Table S1: STROBE Statement—Checklist of items that should be included in reports of cohort studies; Table S2: Spearman correlation matrix of the fourteen preoperative blood-derived indices (*n* = 142); Table S3: Influence diagnostics for the NRBC% coefficient in the pre-specified model.

## Abbreviations

The following abbreviations are used in this manuscript:

ALT	Alanine Transaminase
APRI	Aspartate Transaminase-to-Platelet Ratio Index
AST	Aspartate Transaminase
AUC	Area Under the Curve
CAD	Coronary Artery Disease
CI	Confidence Interval
CRP	C-Reactive Protein
DFS	Disease-Free Survival
DM	Diabetes Mellitus
EPV	Events Per Variable
FIB-4	Fibrosis Index Based on Four Factors
FIGO	International Federation of Gynecology and Obstetrics
GLR	Glucose-to-Lymphocyte Ratio
HB	Hemoglobin
HCT	Hematocrit
HR	Hazard Ratio
HT	Hypertension
IBI	Inflammatory Burden Index
ICPI	Inflammation-Combined Prognostic Index
IG	Immature Granulocyte
LMR	Lymphocyte-to-Monocyte Ratio
LVSI	Lymphovascular Space Invasion
MLR	Monocyte-to-Lymphocyte Ratio
NLR	Neutrophil-to-Lymphocyte Ratio
NRBC	Nucleated Red Blood Cell
OS	Overall Survival
PIV	Pan-Immune–Inflammation Value
PLR	Platelet-to-Lymphocyte Ratio
PLT	Platelet Count
SD	Standard Deviation
SII	Systemic Immune–Inflammation Index
SIRI	Systemic Inflammation Response Index
STROBE	Strengthening the Reporting of Observational Studies in Epidemiology
ULN	Upper Limit of Normal
WBC	White Blood Cell
