# Supplementary material for: Prognostic Value of Preoperative Inflammatory, Hematological, and Metabolic Blood Indices in Endometrioid-Type Endometrial Cancer: A Retrospective Cohort Study
Source: Medicina (Kaunas). 2026 Sep 14;62(9):1767. doi: 10.3390/medicina62091767 (PMC13609171; doi:10.3390/medicina62091767)
Supplement: Supplementary file 1 [file medicina-62-01767-s001.zip › Table S2 Spearman matrix.pdf]

Table S2. Spearman correlation matrix of the fourteen preoperative blood-derived indices (n = 142)

Manuscript ID: medicina-4489729

Prognostic Value of Preoperative Inflammatory, Hematological and Metabolic Blood Indices in Endometrioid-Type Endometrial Cancer: A Retrospective Cohort Study

	NLR	PLR	MLR	LMR	SII	SIRI	PIV	IBI	ICPI	GLR	APRI	FIB-4	NRBC%	IG%
**NLR**	1.00													
**PLR**	0.53	1.00												
**MLR**	0.58	0.40	1.00											
**LMR**	−0.58	−0.40	**−1.00**	1.00										
**SII**	**0.85**	0.66	0.44	−0.44	1.00									
**SIRI**	**0.80**	0.26	**0.74**	**−0.74**	**0.75**	1.00								
**PIV**	**0.70**	0.40	0.59	−0.59	**0.87**	**0.90**	1.00							
**IBI**	0.53	0.15	0.24	−0.24	0.56	0.55	0.58	1.00						
**ICPI**	**0.75**	0.49	**0.83**	**−0.83**	0.65	**0.76**	0.67	0.32	1.00					
**GLR**	0.48	0.60	0.36	−0.36	0.31	0.14	0.06	0.04	0.39	1.00				
**APRI**	−0.05	−0.19	0.22	−0.22	−0.35	−0.08	−0.33	−0.20	0.07	0.23	1.00			
**FIB-4**	−0.02	−0.30	0.19	−0.19	−0.38	−0.05	−0.35	−0.17	0.04	0.19	**0.75**	1.00		
**NRBC%**	−0.05	0.03	0.11	−0.11	−0.08	−0.06	−0.09	−0.11	0.00	0.12	0.11	0.11	1.00	
**IG%**	0.20	−0.15	0.05	−0.05	0.12	0.26	0.18	0.20	0.11	0.00	−0.15	−0.02	0.01	1.00

Coefficients are Spearman rank correlations computed on pairwise complete observations and rounded to two decimal places. Indices are ordered by biological category: inflammatory and immune-cell indices (NLR to ICPI), metabolic and hepatic indices (GLR to FIB-4), and analyser-reported haematological parameters (NRBC%, IG%). Only the lower triangle is shown, the matrix being symmetric. Cells are shaded where the absolute coefficient is at least 0.70 (darker) or at least 0.40 (lighter).

IBI and its component C-reactive protein were available in 76 of 142 patients (53.5%); coefficients involving IBI are therefore based on that subset. All other coefficients are based on the full cohort.

LMR and MLR are exact reciprocals by construction, which is why their rank correlation is −1.00. FIB-4 and APRI share aspartate transaminase and platelet count (rho = 0.75), and SIRI and MLR share the monocyte-to-lymphocyte term (rho = 0.74); one representative marker was retained from each of these pairs for multivariable modeling, as described in Section 2.6. SII and PIV share three of four components (rho = 0.87). ICPI, calculated with the binary scoring of its source publication, correlates most closely with MLR and LMR (rho = 0.83 and −0.83) and only moderately with PLR; the coefficient for that pair is 0.495, which appears as 0.49 in the matrix above after rounding to two decimal places and is quoted as 0.50 in the main text. NRBC% and IG% correlate weakly with every ratio-based index (maximum absolute coefficient 0.26), indicating that they carry information distinct from the conventional inflammatory ratios.

APRI: aspartate transaminase-to-platelet ratio index; FIB-4: fibrosis index based on four factors; GLR: glucose-to-lymphocyte ratio; IBI: inflammatory burden index; ICPI: inflammation-combined prognostic index; IG: immature granulocyte; LMR: lymphocyte-to-monocyte ratio; MLR: monocyte-to-lymphocyte ratio; NLR: neutrophil-to-lymphocyte ratio; NRBC: nucleated red blood cell; PIV: pan-immune-inflammation value; PLR: platelet-to-lymphocyte ratio; SII: systemic immune-inflammation index; SIRI: systemic inflammation response index.
