# Supplementary material for: Prognostic Value of Preoperative Inflammatory, Hematological, and Metabolic Blood Indices in Endometrioid-Type Endometrial Cancer: A Retrospective Cohort Study
Source: Medicina (Kaunas). 2026 Sep 14;62(9):1767. doi: 10.3390/medicina62091767 (PMC13609171; doi:10.3390/medicina62091767)
Supplement: Supplementary file 1 [file medicina-62-01767-s001.zip › Table S3 Influence diagnostics.pdf]

Table S3. Influence diagnostics for the NRBC% coefficient in the pre-specified model

Manuscript ID: medicina-4489729

Prognostic Value of Preoperative Inflammatory, Hematological and Metabolic Blood Indices in Endometrioid-Type Endometrial Cancer: A Retrospective Cohort Study

Panel A. Reference model M1 (age category, histologic grade, FIGO stage, NRBC%); n = 142, 34 events

Covariate	Hazard ratio (95% CI)	p
Age ≥65 versus <65 years	2.24 (1.11–4.53)	0.024
Histologic grade 2 versus 1	3.41 (1.56–7.48)	0.002
Histologic grade 3 versus 1	2.19 (0.64–7.46)	0.212
FIGO stage ≥II versus I	0.95 (0.40–2.28)	0.912
NRBC%, per 1% increase	3.42 (1.46–8.04)	0.005
Harrell’s concordance index	0.679 (SE 0.047)	—

Panel B. Summary influence statistics for the NRBC% coefficient

Statistic	Value
Standard error of the NRBC% coefficient	0.436
DFBETA, range across all 142 observations	−0.065 to +0.064
Scaled DFBETA (DFBETA divided by the standard error)	−0.149 to +0.146
Deviance residual, range	−1.53 to +2.74
Observations with |deviance residual| > 2	7 of 142

Panel C. The ten observations with the largest scaled DFBETA for the NRBC% coefficient

Rank	NRBC%	Vital status	Follow-up (months)	DFBETA	Scaled DFBETA
1	0.00	Died	5	−0.0650	−0.149
2	0.00	Died	8	−0.0650	−0.149
3	0.00	Died	11	−0.0650	−0.149
4	0.50	Died	24	+0.0637	+0.146
5	0.60	Alive	71	−0.0537	−0.123
6	0.00	Died	2	−0.0490	−0.113
7	0.00	Died	10	−0.0487	−0.112
8	0.00	Died	20	−0.0472	−0.108
9	0.00	Died	13	−0.0407	−0.093
10	0.50	Died	13	+0.0394	+0.090

Panel D. All observations with |deviance residual| > 2

NRBC%	Vital status	Follow-up (months)	Deviance residual
0.00	Died	1	+2.69
0.00	Died	2	+2.59
0.00	Died	5	+2.74
0.00	Died	8	+2.63
0.00	Died	10	+2.17
0.00	Died	11	+2.41

NRBC%	Vital status	Follow-up (months)	Deviance residual
0.00	Died	24	+2.01

Panel E. Leave-one-out analysis (the model refitted 142 times, omitting one patient each time)

Quantity	Value
NRBC% hazard ratio, range across the 142 refits	2.14 to 3.66
NRBC% p value, range across the 142 refits	0.002 to 0.655
Refits in which p ≥ 0.05	1 of 142
Patient omitted in that refit	NRBC% 2.50, died
Resulting estimate in that refit	HR 2.14 (0.08–60.40), p = 0.655

Panel F. Sensitivity analyses excluding the upper tail of the NRBC% distribution

Analysis	n	Events	NRBC% HR (95% CI)	p
Full cohort	142	34	3.42 (1.46–8.04)	0.005
Excluding the single highest value (NRBC% 2.50)	141	33	2.14 (0.08–60.40)	0.655
Excluding all values above 0.30% (5 patients)	137	31	0.03 (0.00–30.22)	0.324

Notes

The pre-specified clinical model M0 comprises age category, histologic grade and FIGO stage; M1 adds NRBC% as a continuous term. All diagnostics were computed on M1. Hazard ratios for NRBC% are expressed per one percentage point, an increment that exceeds the range observed in most patients; this is the reason for the width of the confidence intervals reported in Panels E and F.

Panel B. DFBETA is the change in the estimated coefficient when a single observation is omitted, obtained from the approximation implemented in the survival package. Scaling by the standard error of the coefficient expresses that change in units of its own uncertainty; values of this magnitude, all below 0.15, indicate that no single observation is influential by conventional criteria.

Panels C and D. Observations are identified by their NRBC% value, vital status and follow-up time rather than by any patient identifier. In Panel C every observation with a negative DFBETA is a patient with undetectable NRBC% who died early, and the effect of these patients is to reduce the coefficient rather than to inflate it. All seven observations in Panel D likewise had undetectable NRBC% and died within 24 months, indicating that the cases the model fits least well are early deaths that NRBC% does not account for, not patients with extreme marker values.

Panels E and F. Only one patient had an NRBC% above 0.60, so the analysis excluding the single highest value and the analysis excluding values above 0.60 are identical. When that patient is removed the estimate does not move towards the null but loses precision, and when all five patients above 0.30% are removed the remaining variation in NRBC% is too small to support any estimate. The coefficient is therefore not the product of an aberrant leverage point, but the range over which it can be estimated is created almost entirely by a small number of patients at the top of the distribution.

CI: confidence interval; HR: hazard ratio; NRBC: nucleated red blood cell; SE: standard error. Analyses were performed in R version 4.6.1 using the survival package.
